# The Effect of Methylcholanthrene on the Incidence of Lung Tumours in Two Inbred Strains of Mice and their Reciprocal Hybrids

**DOI:** 10.1038/bjc.1954.50

**Published:** 1954-09

**Authors:** E. W. Miller, F. C. Pybus


### THE EFFECT OF METHYLCHOLANTHRENE ON THE INCIDENCE OF LUNG TUMOURS IN TWO INBRED STRAINS OF MICE AND THEIR RECIPROCAL HYBRIDS.

E. W. MILLER AND F. C. PYBUS.

*From the J. H. Burn Research Laboratory, Royal Victoria Infirmary, Newcastle-upon-Tyne.*

Received for publication May 28, 1954.

### METHODS.

RECIPROCAL crosses were made between the two inbred strains of mice, NBT and CBA, and the offspring were inbred for 12 generations. Half the members of each $F_1$ litter received one subcutaneous injection each of 1·0 mg. methylcholanthrene in 0·1 c.c. sesame oil at the age of 2 months, and their offspring were inbred for 11 more generations, the mice of $F_1$ to $F_{10}$ being injected. Details of the experiment have been described (Miller and Pybus, 1954). The injected $F_1$ mice and their descendants were designated the M/CBA/NBT and M/NBT/CBA strains (or MCN and MNC, for short) to distinguish them from the control CBA/NBT and NBT/CBA hybrids (CN and NC, for short), the maternal strain being named first. The last 2 generations, $F_{11}$ and $F_{12}$, of the MCN and MNC strains were not injected, neither were certain litters of each cross from $F_5$ onwards whose parents, at an early age, developed local tumours ; these constituted the *uninjected* MCN and MNC groups.

Mice from the inbred parent strains were also injected, and subsequently bred from for one (uninjected) generation only. The injected mice formed the MCBA and MNBT groups, and their untreated offspring the MCBA $F_1$ and MNBT $F_1$ groups.

The main object of the experiment had been to obtain lines of hybrids resistant to the development of local tumours, in order to study the incidence of remote tumours, as had been done by Strong (1940, 1945), but no such resistant lines appeared.

In the following presentation of the results, data are given for the lung tumour incidence in the parent inbred strains and in the reciprocal hybrids. Within each group the tumour incidence in males and females are compared, as are also the ages at which tumours appeared in the two sexes, and the incidences and tumour ages of spontaneous tumours (in untreated mice) are examined and compared with the corresponding values for induced tumours (in mice injected with methylcholanthrene). Comparison is also made, for spontaneous and for induced tumours, between the 2 pure strains, and between the reciprocal hybrids.

The figures are based on the effective number of mice in each group (i.e., the number surviving at the time of discovery of the earliest lung tumour in that group). It is appropriate here to point out that, in common with other neoplasms of internal organs, the existence of a lung tumour is discovered only at death. As it is usually of slow growth and is only rarely the cause of death, the tumour may have been present for a considerable time. Thus, in a comparison between average tumour ages in the various groups, if mice of one sex or of one strain are naturally shorter-lived, their average tumour age will be proportionately earlier. In the injected groups, where so many animals were killed young on account of local tumours, the " lung tumour age " (i.e., the age at which the tumours were discovered) probably corresponded more closely with the actual age of lung tumour development.

The lung tumours were of the normal type of adenoma or carcinoma so often described elsewhere ; they varied in size from minute nodules to large growths occupying a whole lobe, and in the latter case could be the cause of death. The majority appeared to be of very slow growth.

Throughout this paper the word " tumour," without qualification, is to be understood to refer to lung tumour.

### RESULTS.

### 1. *Parent Pure Strains.*

A. *Controls.*

During the period of the experiment, mice from the 2 inbred strains were observed as controls ; these came from Generations 25 to 36 of the CBA, and Generations 28 to 40 of the NBT. The tumour data are shown in Table I. In neither strain was the difference between the incidences in males and females significant.[1*] The difference between the strains, 6·0 per cent incidence in CBA compared with 2·1 per cent in NBT, was significant.[2]

Although the incidence was the same in both sexes, there was a significant difference in both strains between the average tumour ages in males and females,[3] on account of which it was impossible to take an average tumour age for each strain as a whole and the sexes were compared separately (Table I). The tumorous females of both strains died about 4 months later than the males.

Although the NBT mice were the more resistant, their average tumour age was over 10 months less than that of the CBA strain. The CBA mice (bred by Strong for long life (Strong, 1936)) lived to a much greater age than the NBT, and in both strains the females lived longer than the males ; hence the tumours were found later in the females, and later in the CBA mice.

The incidences in both strains were low compared with those reported for Strain A (70–89 per cent) and Strain C (20–30 per cent) by Shimkin (1940*a*). The CBA mice were about as susceptible as the " moderately susceptible " C3H (7·0 per cent (Shimkin, 1940*a*)), while the incidence in the NBT mice was slightly higher than that of the " relatively resistant " $C_{57}$Bl strain (Shimkin, 1940*a*).

No record was kept of the number of spontaneous lung nodules per mouse in the pure strains, but the impression is that such tumours were usually solitary in both strains, but varied in size as already stated.

B. *Injected pure strains.*

The effect of methylcholanthrene was to raise the tumour incidence very greatly in both strains (Table I).

Out of 100 MCBA mice only 13 females and 17 males survived to the age of 12 months (earliest tumour age) ; 9 females and 8 males had tumours. The

* [1] and following numbers in this series refer to the notes on p. 484.

apparent sex-difference was not significant[4] and the total incidence of 56·7 per cent was almost 10 times that of the controls.

In the MNBT group, out of 131 mice only 49 females and 17 males survived to the earliest tumour age of 7 months, and of these 8 females and 2 males had tumours. Again the sex-difference was not significant[5] and the total incidence of 15·2 per cent was more than 7 times the control incidence.

The difference between the strain-incidences was significant,[6] MCBA mice being the more susceptible to this effect of the carcinogen. This is in agreement with the results for other inbred strains (Lynch, 1927 ; Andervont, 1937*a* ; Shimkin, 1940*a*) in which it was shown that susceptibility to induced tumours parallels that to spontaneous tumours.

The average tumour ages in males and females in the MCBA group did not differ significantly and both were several months earlier than in the controls (Table I).

TABLE I.—*The Incidence of Lung Tumours in the Two Inbred Strains of Mice, CBA and NBT, in the Methylcholanthrene-treated M/CBA and M/NBT, and in the Untreated Offspring of the Latter, M/CBA $F_1$ and M/NBT $F_1$.*

| Strain. | | Total effective number of mice. | Tumorous mice. Number. | Percentage. | | Average age at death (months). | | σ. | | Earliest tumour (months). | Latest tumour (months). |
|---|---|---|---|---|---|---|---|---|---|---|---|
| CBA | females | 243 | 19 | 7·8 | 5·95 | 26·3 | | 2·7 | | 14 | 31 |
| | males | 261 | 11 | 4·2 | | 22·5 | | 4·1 | | | |
| NBT | females | 287 | 5 | 1·7 | 2·1 | 16·0 | | 3·2 | | 5 | 20 |
| | males | 334 | 8 | 2·4 | | 11·9 | | 3·9 | | | |
| M/CBA | females | 13 | 9 | 69·2 | 56·7 | 16·7 | 17·0 | 3·5 | 3·8 | 12 | 24 |
| | males | 17 | 8 | 47·1 | | 17·4 | | 4·1 | | | |
| M/NBT | females | 49 | 8 | 16·3 | 15·2 | 14·8 | 13·7 | 2·6 | 3·3 | 7 | 18 |
| | males | 17 | 2 | 11·8 | | 9·5 | | 4·3 | | | |
| M/CBA | females | 167 | 8 | 4·7 | 4·9 | 27·6 | | 4·4 | | 17 | 34 |
| | males | 137 | 7 | 5·1 | | 21·3 | | 3·0 | | | |
| M/NBT $F_1$ | females | 73 | 8 | 11·0 | 7·0 | 16·3 | 15·8 | 3·1 | 3·4 | 9 | 19 |
| | males | 84 | 3 | 3·6 | | 14·3 | | 3·7 | | | |

There was no significant sex-difference in tumour age in the MNBT mice either, and although the average tumour age in each sex was less than that in the controls the difference was not significant.

In the absence of a sex-difference in tumour age, the ages for the two sexes could be averaged in each strain, and the differences of 3·3 months between the two injected groups was significant.[7]

It is impossible to be certain, under the conditions of this experiment, whether the carcinogen actually induced the tumours earlier or whether, as explained in the introduction, they were merely seen earlier. For an accurate comparison an equal number of control mice would have to be killed at the same time as the injected animals.

The development of induced multiple lung nodules is a well-known test of the tumour-susceptibility of a strain (Andervont, 1937*a* ; Lynch, 1940 ; Shimkin,

1940*a*; Heston, 1940). In the MCBA group, 2 of the 17 mice with induced tumours had solitary nodules, the remainder having 2 or more, an incidence of 11·8 per cent with solitary nodules. In the MNBT group, 6 of the 10 cases of induced tumours were solitary occurrences, an incidence of 60 per cent. The numbers in each class were small but the difference between the strains was significant [8] and confirmed the difference in susceptibility.

C. *The uninjected offspring* ($F_1$) *of each injected pure strain.*

There was no significant sex-difference in the tumour incidence in either group and the totals of 4·9 per cent in 304 MCBA $F_1$ mice and 7·0 per cent in 157 MNBT $F_1$ mice were in agreement. The MCBA $F_1$ tumour incidence did not differ significantly from that of 5·95 per cent in the CBA controls (Table I), but the MNBT $F_1$ tumour incidence showed a significant increase over the figure of 2·1 per cent in the NBT controls. [9]

This latter increase may have a genetical origin. Two pairs of injected MNBT mice (belonging to $F_{28}$ and $F_{29}$ of the strain), none of which showed a tumour, each had 3 tumorous " $F_1$ " offspring, 3 out of 10 mice (equivalent to $F_{29}$ in the pure strain) from the first pair, and 3 out of 4 mice (equivalent to $F_{30}$) from the second. These two tumorous families both had close relations bearing tumours; in the first instance there were 2 spontaneous tumours in 9 $F_{27}$ mice, in the second there were 3 induced tumours amongst the 26 injected $F_{29}$ sibs of the injected parents. These two families may therefore have been more susceptible to lung tumours than were the remainder, for some reason such as a possible mutation in the line prior to treatment. The remaining 5 tumour mice of the 11 in MNBT $F_1$ were scattered singly and apparently at random amongst the families from the other 25 pairs of injected parents.

In the absence of a corresponding increase in tumour incidence in the MCBA $F_1$ group, there seems no reason to postulate the transmission of a carcinogenic effect from the injected parents.

An analysis of the tumour ages in the $F_1$ groups showed a sex-difference in MCBA but not in MNBT; [10] in the latter, the female tumour age followed the usual trend and was later than that of the males but not significantly so (owing to the small number of tumour males). The average tumour age in MCBA $F_1$ was many months later for both males and females compared with the corresponding MNBT $F_1$ animals. There was no significant difference in tumour age in either strain between the control and $F_1$ groups.

*Summary.*

The CBA strain was more susceptible both to spontaneous and to induced tumours than the NBT. There was no sex-difference in either strain in the incidence of either type of tumour. The average spontaneous tumour age was much later in the females than in the males in both strains and much later in the CBA than in the NBT; this difference could be due to the longer life of females than males and of CBA mice than NBT. The sex-difference in tumour age disappeared in the case of induced tumours but the strain-difference persisted, although much reduced compared with the controls. The tumour incidence and average tumour age in the generation of uninjected mice raised from the treated MCBA animals reverted to the control values, but in the MNBT $F_1$ group the incidence was raised to the CBA level, probably owing to the inclusion of two unusually susceptible families. There were more mice with multiple lung nodules in the MCBA group than in the MNBT.

### 2. *Hybrid Strains* ($F_1$ *and* $F_2$).

The first two hybrid generations will be analysed separately. Analysis of the total spontaneous lung tumour incidence in all 12 generations of the reciprocal hybrids seemed to show that the NC mice were more susceptible than the CN (Miller and Pybus, 1952) ; this was not confirmed by an examination of the data for the first two generations.

A. *Spontaneous tumours.*

(1) *First hybrid generation.*—There was no significant difference between tumour incidences in the reciprocal hybrids (36·4 per cent in CN $F_1$ and 38·4 per cent in NC $F_1$) or in the two sexes in each group (Table II).

The $F_1$ mice lived longer than did those of later generations owing to hybrid vigour, and the average $F_1$ tumour ages were greater than the strain averages, but only in the CN males and the NC females were the differences in tumour age between $F_1$ and the strain large enough to be significant.[11] The earliest CN $F_1$ tumour was found at 18 months and the earliest NC $F_1$ tumour at 16 months.

Tumours were found significantly earlier in the males of each group.[12] Comparing the two groups, the average tumour ages for the females were in agreement, but the difference (2·7 months) between the males was significant.[13] This could be explained by the longer life of all CN $F_1$ males (23·2 months) compared with all NC $F_1$ males (19·7 months), tumours being found earlier in the latter.

(2) *Second hybrid generation.*—Again there was no sex-difference in tumour incidence, neither was the difference in incidence between the two groups significant (23·9 per cent in CN $F_2$, 18·7 per cent in NC $F_2$) (Table II).

There was no significant sex-difference in average tumour age in NC $F_2$, but, in CN $F_2$, tumours were found 3 months later in the males than in the females, a significant difference. Comparing the 2 groups, the average tumour ages for

TABLE II.—*Incidence of Spontaneous Lung Tumours in the First Two Generations of Two Reciprocal-hybrid Strains of Mice*

| Strain and generation. | Number of mice. Total. | Number of mice. Effective. | Tumorous mice. Number. | Tumorous mice. Percentage. | Tumorous mice. Average age at death (months). | Tumorous mice. σ. | Earliest tumour (months). | Latest tumour (months) |
|---|---|---|---|---|---|---|---|---|
| N $F_1$ females | 58 | 52 | 17 | 32·7 } 36·4 | 26·6 | 4·6 } | 18 | 34 |
| males | 54 | 47 | 19 | 40·4 | 23·9 | 2·6 | | |
| $F_2$ females | 145 | 127 | 26 | 20·5 } 23·9 | 24·8 | 2·7 } | 16 | 30 |
| males | 116 | 99 | 28 | 28·3 | 21·8 | 2·7 | | |
| C $F_1$ females | 58 | 56 | 18 | 32·1 } 38·4 | 26·7 | 4·6 } | 16 | 35 |
| males | 46 | 30 | 15 | 50·0 | 21·2 | 2·7 | | |
| $F_2$ females | 109 | 105 | 24 | 22·9 } 18·7 | 25·0 | 3·3 } | 12 | 30 |
| males | 129 | 114 | 17 | 14·9 | 22·9 | 4·1 | | |

females were in agreement, as were also those for males. The earliest CN $F_2$ tumour was found at 16 months and the earliest NC $F_2$ tumour at 12 months.

Comparing $F_1$ and $F_2$, the NC $F_2$ incidence was significantly lower than the NC $F_1$,[14] but the difference between CN $F_1$ and $F_2$ was barely significant.[15] The mean tumour ages for the 2 generations were in agreement except for the CN males.[16] As a larger proportion of CN $F_2$ males died at an earlier age than did those of $F_1$, there was a greater opportunity for the discovery of early tumours in CN $F_2$.

The lower incidences in the $F_2$'s may be an indication of the segregation of factors concerned with tumour susceptibility, but there is another possible explanation which came to light when the data for induced tumours in the 2 generations were examined.

B. *Induced tumours.*

(1) *First hybrid generation.*—There was no significant difference between tumour incidences in the two sexes or in the reciprocal hybrids (46·5 per cent in MCN $F_1$ and 37·8 per cent in MNC $F_1$) (Table III). Neither was there a significant difference between the incidences of spontaneous and induced tumours in $F_1$. This latter result was unexpected and will be referred to again in the discussion; briefly, it raised the question of possible contamination of some $F_1$ mice with the carcinogen.

TABLE III.—*Incidence of Induced Lung Tumours in the First Two Generations of Two Reciprocal-hybrid Strains of Mice.*

| Strain and generation. | | Number of mice. Total. | Effective. | Tumorous mice. Number. | Percentage. | | Average at at death (months). | σ. | Earliest tumour (months). | Latest tumour (months). |
|---|---|---|---|---|---|---|---|---|---|---|
| MCN $F_1$ | females | 47 | 34 | 18 | 52·9 | 46·5 | 17·3 | 4·9 | 8 | 28 |
| | males | 50 | 37 | 15 | 40·5 | | 19·9 | 4·3 | | |
| $F_2$ | females | 134 | 96 | 45 | 46·9 | 46·2 | 15·5 | 5·5 | 7 | 28 |
| | males | 120 | 75 | 34 | 45·3 | | 13·9 | 4·5 | | |
| MNC $F_1$ | females | 51 | 38 | 15 | 39·5 | 37·8 | 17·8 | 6·0 | 7 | 32 |
| | males | 54 | 36 | 13 | 36·1 | | 20·6 | 6·1 | | |
| $F_2$ | females | 122 | 108 | 39 | 36·1 | 35·6 | 14·6 | 4·9 | 6 | 26 |
| | males | 125 | 114 | 40 | 35·1 | | 15·7 | 5·1 | | |

The earliest MCN $F_1$ tumour was found at 8 months and the earliest MNC $F_1$ tumour at 7 months. Mean tumour ages in the injected mice were not significantly different in the two sexes or between the reciprocal hybrids, and were much lower than in the controls except in the MNC $F_1$ males; NC $F_1$ males died relatively young. Mean tumour ages were rather later than for the whole 10 generations of injected mice (*cf.* Table XVI), but significantly so only for the males (5·8 months later in MCN $F_1$, 6·9 months later in MNC $F_1$).[17]

(2) *Second hybrid generation.*—The induced tumour incidences in the reciprocal $F_2$'s were not significantly different (46·2 per cent in MCN $F_2$, 35·6 per cent in MNC $F_2$) (Table III), and there was no sex-difference.

There was no difference between mean tumour ages in the two sexes in either strain, neither did the tumour ages differ between the reciprocal hybrids.

The incidence of induced $F_2$ tumours was significantly higher than that of spontaneous $F_2$ tumours in the reciprocal crosses,[18] and induced tumours were found much earlier than spontaneous tumours in the $F_2$ mice.

Comparing $F_1$ and $F_2$, the incidences of induced tumours in the 2 generations were in agreement. Between $F_1$ and $F_2$ females the difference in induced-tumour age was not significant, but the difference between the males was significant,[19] owing to more of the $F_2$ males dying young and tumours being seen earlier.

C. *Multiple nodules.*

The post-mortem records were analysed for the occurrence of multiple nodules (Table IV). It was found that the majority of tumorous control mice in $F_1$ and $F_2$ of the reciprocal crosses had single lung nodules. The differences between the 2 strains and between the 2 generations in the same strain were not significant. In the injected $F_1$ and $F_2$ groups, the majority of the tumorous mice had multiple nodules (2 or more, the exact number over 2 not being stated in the records). The differences between the injected reciprocal hybrids, and between $F_1$ and $F_2$ in each injected group, were not significant, but the differences between the controls and the injected mice were significant.[20]

TABLE IV.—*Proportion of Tumour Mice Bearing Solitary Lung Nodules, in Methylcholanthrene-treated Animals and their Controls in Two Reciprocal-hybrid Crosses.*

| Strain. | Generation. | Number of tumour mice. | Mice with single lung nodules. Number. | Percentage. | Mice with two or more lung nodules. Number. | Percentage. |
|---|---|---|---|---|---|---|
| CN | $F_1$ | 36 | 26 | 72·2 | 10 | 27·8 |
| | $F_2$ | 54 | 45 | 83·3 | 9 | 16·7 |
| | $F_1$–$F_{12}$ | 142 | 109 | 76·8 | 33 | 23·2 |
| MCN uninjected | $F_5$–$F_{12}$ | 40 | 31 | 77·5 | 9 | 22·5 |
| MCN injected | $F_1$ | 33 | 8 | 24·2 | 25 | 75·8 |
| | $F_2$ | 79 | 27 | 34·2 | 52 | 65·8 |
| | $F_1$–$F_{10}$ | 276 | 100 | 36·6 | 176 | 63·4 |
| NC | $F_1$ | 33 | 27 | 81·8 | 6 | 19·2 |
| | $F_2$ | 41 | 30 | 73·2 | 11 | 26·8 |
| | $F_1$–$F_{12}$ | 173 | 132 | 76·3 | 41 | 23·7 |
| MNC uninjected | $F_5$–$F_{12}$ | 169 | 155 | 91·7 | 14 | 8·3 |
| MNC injected | $F_1$ | 28 | 12 | 42·9 | 16 | 57·1 |
| | $F_2$ | 79 | 25 | 31·6 | 54 | 68·4 |
| | $F_1$–$F_{10}$ | 222 females | 76 | 34·2 | 146 | 65·8 |
| | | 165 males | 79 | 47·9 | 86 | 52·1 |

3. *Hybrid Strains* (*all generations*).

A. *Spontaneous tumours.*

The data for spontaneous lung tumour incidence and ages in all generations of the reciprocal hybrids are given in Tables V to XIII. The incidences are based on the number of mice living at the time of the earliest tumour in each group, so that the incidences in $F_1$ and $F_2$ (Tables V and IX), based on the numbers alive at 11 months, are less than those given in Table II in which the incidences (for strict comparison of the individual generations) were calculated on the effective number of mice in each generation, *i.e.*, those alive at 18 and 16 months in the $F_1$'s and at 16 and 12 months in the $F_2$'s. Tables V and IX give the totals for all 12 generations and also for $F_2$ to $F_{12}$, *i.e.*, excluding the reciprocal $F_1$'s on account of the doubts mentioned above ; the totals for $F_5$ to $F_{12}$ are given for comparison with the uninjected MCN and MNC groups.

(1) *CN and uninjected MCN hybrids* (*Tables V to VIII*).—There was no significant sex-difference in tumour incidence in either group, so the combined incidences for both sexes are given (Tables V and VI). The difference between the incidences in the 2 groups, 13·6 per cent in CN (or 11·3 per cent without $F_1$) and 5·6 per cent in uninjected MCN, was significant.[21] As the uninjected MCN animals came from Generations 5 to 12, the figures for these generations were compared. The totals of 28 tumours in 556 CN mice (5·0 per cent) and 40 tumours in 720 MCN uninjected mice (5·6 per cent) were in agreement. The high incidence in the whole CN strain was due to the high incidence in the early generations, from which there was a marked fall to a moderately low incidence with considerable fluctuation about

TABLE V.—*Incidence of Spontaneous Lung Tumours in each Generation of CN Hybrids.*

| Generation. | Number of mice. Total in experiment. | Surviving to tumour age. | Tumorous mice. Number. | Percentage. |
|---|---|---|---|---|
| $F_1$ | 112 | 107 | 36 | 33·6 |
| $F_2$ | 261 | 248 | 54 | 21·8 |
| $F_3$ | 76 | 66 | 16 | 24·2 |
| $F_4$ | 83 | 65 | 8 | 12·3 |
| $F_5$ | 71 | 44 | 4 | 9·1 |
| $F_6$ | 53 | 33 | 2 | 6·1 |
| $F_7$ | 63 | 41 | 4 | 9·8 |
| $F_8$ | 96 | 68 | 2 | 2·9 |
| $F_9$ | 100 | 48 | 7 | 14·6 |
| $F_{10}$ | 113 | 63 | 2 | 3·2 |
| $F_{11}$ | 130 | 51 | 1 | 2·0 |
| $F_{12}$ | 275 | 208 | 6 | 2·9 |
| $F_1$–$F_{12}$ | 1433 | 1042 | 142 | 13·6 |
| $F_2$–$F_{12}$ | 1321 | 935 | 106 | 11·3 |
| $F_5$–$F_{12}$ | 901 | 556 | 28 | 5·0 |

TABLE VI.—*Incidence of Spontaneous Lung Tumours in each Generation of Uninjected MCN Hybrids.*

| Generation. | Number of mice. Total in experiment. | Surviving to tumour age. | Tumorous mice. Number. | Percentage. |
|---|---|---|---|---|
| $F_5$ | 92 | 86 | 10 | 11·6 |
| $F_6$ | 12 | 5 | 1 | 20·0 |
| $F_7$ | 44 | 40 | 2 | 5·0 |
| $F_8$ | 81 | 69 | 4 | 5·8 |
| $F_9$ | 107 | 75 | 3 | 4·0 |
| $F_{10}$ | 109 | 78 | 7 | 9·0 |
| $F_{11}$ | 222 | 149 | 9 | 6·0 |
| $F_{12}$ | 275 | 218 | 4 | 1·8 |
| $F_5$–$F_{12}$ | 942 | 720 | 40 | 5·6 |

that figure in the last 8 generations of both groups, doubtless due to a segregation of factors for susceptibility and a chance breeding of the less susceptible lines.

Tumours were found much earlier in the uninjected MCN mice than in the CN group as a whole (Table VII). Taking the last 8 generations of the CN hybrids, the difference between the males was reversed owing to the shorter life ofCN males in the later generations. Table VIII gives the mean ages at death of all the CN and uninjected MCN mice ; the males died earlier than the females, the difference within each group being significant[22], [23]. The differences between the 2 groups were also significant.[24] The earlier tumour age in the uninjected MCN group was therefore due to the mice of that group dying earlier and the tumours being found sooner.

TABLE VII.—*Ages at Death of Tumour Mice in Control CN and Uninjected MCN Hybrids.*

| Strain. | Generation. | Tumour females. Number. | Average age at death (months). | $\sigma$. | Tumour males. Number. | Average age at death (months). | $\sigma$. | Earliest tumour (months). | Latest tumour (months). |
|---|---|---|---|---|---|---|---|---|---|
| CN | $F_1$–$F_{12}$ | 76 | 24·7 | 4·5 | 66 | 22·0 | 3·8 | 11 | 34 |
| CN | $F_5$–$F_{12}$ | 21 | 23·3 | 5·1 | 7 | 15·3 | 2·2 | 12 | 32 |
| MCN uninjected | $F_5$–$F_{12}$ | 25 | 16·3 | 3·2 | 15 | 17·4 | 3·9 | 11 | 24 |

TABLE VIII.—*Ages at Death of Control CN and Uninjected MCN Mice.*

| Strain. | Generation. | Females. Total in experiment. | Average age at death (months). | $\sigma$. | Males. Total in experiment. | Average age at death (months). | $\sigma$. |
|---|---|---|---|---|---|---|---|
| CN | $F_1$–$F_{12}$ | 706 | 19·4 | 7·2 | 727 | 13·5 | 6·6 |
| MCN uninjected | $F_5$–$F_{12}$ | 438 | 13·7 | 4·4 | 504 | 12·4 | 4·1 |

(2) *NC and uninjected MNC hybrids.* (*Tables IX to XIII*).—The tumour incidences for the whole 12 generations of NC hybrids (Table IX) were 21·8 per cent in females and 12·9 per cent in males, or 20·6 per cent and 10·4 per cent without $F_1$, both significant sex-differences, [25] and this was the only group in the whole experiment to show a sex-difference in the incidence of spontaneous tumours.

In the uninjected MNC group the tumour incidences in females (9·9 per cent) and males (9·0 per cent) were in agreement and Table X gives the incidences for the two sexes combined. For the corresponding generations, $F_5$ to $F_{12}$, the NC incidences were 17·4 per cent (females) and 8·4 per cent (males). Comparing the 2 groups, the tumour incidences in the females differed significantly[26] but those in the males were in agreement.

For all the NC mice in the experiment, the mean age at death of the males was much less than that of the females (Table XI), the difference of 5·9 months being significant ;[27] 186 males but only 51 females died before the age of 11 months. Table XII shows the result of a simple calculation based on the numbers of mice alive at 11 months and over, whereby it was found (Column 6) that, if the males

TABLE IX.—*Incidence of Spontaneous Lung Tumours in each Generation of NC Hybrids.*

| Generation. | Number of mice. Total. | Number of mice. Effective. | Tumorous mice. Number | Tumorous mice. Percentage. | Females only. Effective number of mice. | Females only. Number with tumours. | Females only. Percentage tumour incidence. | Males only. Effective number of mice. | Males only. Number with tumours. | Males only. Percentage tumour incidence. |
|---|---|---|---|---|---|---|---|---|---|---|
| $F_1$ | 104 | 97 | 33 | 34·0 | 57 | 18 | 31·6 | 40 | 15 | 37·5 |
| $F_2$ | 238 | 220 | 41 | 18·1 | 105 | 24 | 22·9 | 115 | 17 | 14·5 |
| $F_3$ | 108 | 84 | 13 | 15·5 | 54 | 11 | 20·4 | 30 | 2 | 6·7 |
| $F_4$ | 119 | 101 | 23 | 22·8 | 58 | 18 | 31·8 | 43 | 5 | 11·6 |
| $F_5$ | 96 | 82 | 9 | 11·0 | 38 | 7 | 18·4 | 44 | 2 | 4·5 |
| $F_6$ | 83 | 68 | 10 | 14·7 | 43 | 9 | 20·9 | 25 | 1 | 4·0 |
| $F_7$ | 72 | 58 | 8 | 13·8 | 22 | 4 | 18·2 | 36 | 4 | 11·1 |
| $F_8$ | 118 | 84 | 14 | 16·7 | 47 | 10 | 21·3 | 37 | 4 | 10·8 |
| $F_9$ | 83 | 61 | 8 | 13·1 | 33 | 4 | 12·1 | 28 | 4 | 14·3 |
| $F_{10}$ | 83 | 47 | 1 | 2·1 | 27 | 0 | 0·0 | 20 | 1 | 5·0 |
| $F_{11}$ | 52 | 24 | 9 | 37·5 | 21 | 8 | 38·1 | 3 | 1 | 33·3 |
| $F_{12}$ | 56 | 49 | 4 | 8·2 | 27 | 3 | 11·1 | 22 | 1 | 4·5 |
| $F_1$–$F_{12}$ | 1212 | 975 | 173 | 17·7 | 532 | 116 | 21·8 | 443 | 57 | 12·9 |
| $F_2$–$F_{12}$ | 1108 | 878 | 140 | 15·9 | 475 | 98 | 20·6 | 403 | 42 | 10·4 |
| $F_5$–$F_{12}$ | 643 | 473 | 63 | 13·3 | 258 | 45 | 17·4 | 215 | 18 | 8·4 |

TABLE X.—*Incidence of Spontaneous Lung Tumours in each Generation of Uninjected MNC Hybrids.*

| Generation. | Number of mice. Total in experiment. | Number of mice. Surviving to tumour age. | Tumorous mice. Number. | Tumorous mice. Percentage. |
|---|---|---|---|---|
| $F_5$ | 84 | 56 | 13 | 23·2 |
| $F_6$ | 217 | 200 | 22 | 11·0 |
| $F_7$ | 154 | 148 | 9 | 6·1 |
| $F_8$ | 88 | 85 | 9 | 10·6 |
| $F_9$ | 81 | 76 | 5 | 6·6 |
| $F_{10}$ | 163 | 150 | 15 | 10·0 |
| $F_{11}$ | 364 | 327 | 21 | 6·7 |
| $F_{12}$ | 781 | 755 | 75 | 9·9 |
| $F_5$–$F_{12}$ | 1932 | 1797 | 169 | 9·4 |

had lived as long as the females, there would have been 113 tumorous males (a 25·5 per cent incidence), which agrees well with the incidence of 21·8 per cent in females. It is proposed to take the tumour incidence in the females as the true incidence for this group.

TABLE XI.—*Ages at Death of Control NC and Uninjected MNC Mice.*

| Strain. | Generation. | Females. Total in experiment. | Females. Average age at death (months). | Females. σ. | Males. Total in experiment. | Males. Average age at death (months). | Males. σ. |
|---|---|---|---|---|---|---|---|
| NC | $F_1$–$F_{12}$ | 583 | 20·8 | 6·3 | 629 | 14·9 | 6·5 |
| NC | $F_5$–$F_{12}$ | 293 | 17·9 | 5·5 | 350 | 12·5 | 5·0 |
| MNC uninjected | $F_5$–$F_{12}$ | 865 | 19·6 | 5·5 | 1067 | 17·5 | 4·8 |

TABLE XII.—*Calculation of Expected Number of Spontaneous Lung Tumours in NC Male Mice, if the Males had Lived as Long as the Females.*

| Age at death (months). (1) | Percentage of mice alive at the beginning of each month. Females. (2) | Males. (3) | Females. / Males. (4) | Number of tumour males. (5) | Columns 4 × 5. (6) | Number of tumour females.* (7) |
|---|---|---|---|---|---|---|
| 11 | 100·0 | 100·0 | 1·00 | 2 | 2·00 | 0 |
| 12 | 97·4 | 91·2 | 1·07 | 2 | 2·14 | 0 |
| 13 | 95·3 | 86·0 | 1·11 | 2 | 2·22 | 2 |
| 14 | 92·8 | 77·9 | 1·19 | 1 | 1·19 | 1 |
| 15 | 89·8 | 70·9 | 1·27 | 1 | 1·27 | 1 |
| 16 | 85·9 | 66·6 | 1·29 | 3 | 3·87 | 2 |
| 17 | 81·6 | 61·0 | 1·34 | 2 | 2·68 | 2 |
| 18 | 77·8 | 52·8 | 1·47 | 4 | 5·88 | 5 |
| 19 | 72·6 | 44·0 | 1·65 | 7 | 11·55 | 4 |
| 20 | 68·2 | 35·0 | 1·95 | 4 | 7·80 | 7 |
| 21 | 62·6 | 28·7 | 2·18 | 7 | 15·26 | 12 |
| 22 | 53·9 | 24·4 | 2·21 | 4 | 8·84 | 12 |
| 23 | 45·7 | 19·0 | 2·41 | 5 | 12·05 | 11 |
| 24 | 37·8 | 15·8 | 2·39 | 3 | 7·17 | 12 |
| 25 | 30·3 | 13·5 | 2·24 | 6 | 13·44 | 8 |
| 26 | 22·7 | 8·6 | 2·64 | 1 | 2·64 | 9 |
| 27 | 17·7 | 5·0 | 3·54 | 1 | 3·54 | 6 |
| 28 | 13·7 | 2·9 | 4·73 | 2 | 9·46 | 6 |
| 29 | 7·7 | 2·0 | 3·85 | 0 | 0·00 | 6 |
| 30 | 5·3 | 1·6 | 3·31 | 0 | 0·00 | 6 |
| 31 | 3·0 | 1·1 | 2·73 | 0 | 0·00 | 2 |
| 32 | 1·9 | 0·2 | 9·50 | 0 | 0·00 | 0 |
| 33 | 1·3 | 0·0 | ∞ | 0 | 0·00 | 1 |
| 34 | 1·1 | 0·0 | ∞ | 0 | 0·00 | 0 |
| 35 | 0·8 | 0·0 | ∞ | 0 | 0·00 | 1 |
| 36 | 0·2 | 0·0 | ∞ | 0 | 0·00 | 0 |
| | Total number of tumours | | | 57 | 113·00 | 116 |

Table XI shows that the mean ages at death of all the MNC uninjected mice were higher than the corresponding mean ages for NC ($F_5$ to $F_{12}$), the differences being significant for each sex.[28]

Mean tumour ages in the uninjected MNC mice showed no sex-difference (Table XIII). Tumours were found later in the uninjected MNC mice than in the corresponding generations of NC mice, the differences between the 2 groups being significant for each sex.[29]

TABLE XIII.—*Ages at Death of Tumour Mice in Control NC and Uninjected MNC Hybrids.*

| Strain. | Generation. | Tumour females. Number. | Average age at death (months). | σ. | Tumour males. Number. | Average age at death (months). | σ. | Earliest tumour (months). | Latest tumour (months). |
|---|---|---|---|---|---|---|---|---|---|
| NC | $F_1$–$F_{12}$ | 116 | 23·6 | 3·2 | 57 | 20·2 | 3·9 | 11 | 35 |
| | $F_5$–$F_{12}$ | 45 | 20·6 | 3·5 | 18 | 16·4 | 3·3 | 11 | 27 |
| MNC uninjected | $F_5$–$F_{12}$ | 80 | 22·2 | 4·0 | 89 | 21·1 | 3·9 | 9 | 29 |

Since the uninjected MNC mice lived longer than the corresponding generations of NC mice, (as much as 5 months longer in the case of the males), the tumour incidence should have been higher in the former than in the latter if the two groups had been equally susceptible. The very much lower incidence in the uninjected MNC mice denotes that the latter were much less susceptible.

Examination of the heredity charts showed that certain lines of NC hybrids appeared to have fewer cases of lung tumours, sometimes probably owing to earlier deaths of the mice from other causes, but one line in particular had a definitely higher incidence persisting through all 12 generations. It was explained in a previous paper (Miller and Pybus, 1954) that, although the original inbred parents of the hybrids were mostly very closely related within their own strains, there was a possibility of genetic difference between those coming from two lines of the CBA strain. The MNC uninjected mice were, with the exception of 41 $F_5$ animals, all the product of one type of cross (C $\times$ P) as previously explained (Miller and Pybus, 1954). An analysis was made of the offspring of the same cross forming part of the NC group, and for strict comparison Generations 5 to 12 were taken. There were 4 lung tumours in 32 females and 5 in 41 males and, as these incidences were in agreement, the total incidence of 9 in 73 mice (12·3) per cent was used; this was not significantly different from the total incidence in the uninjected MNC group of 169 in 1797 mice (9·4 per cent). This confirms the opinion that the lower incidence in the uninjected MNC group (compared with the total NC group) was due to a genetical difference from the majority of the NC hybrids.

Comparing the reciprocal hybrids CN and NC as a whole, the NC mice appeared to be more susceptible to spontaneous lung tumours, the difference between the incidences in the females being significant,[30] but any real difference between the males was masked by the early death of the majority of NC males. The apparent difference between the two groups seems to be due to a segregation of more resistant lines in the later CN generations and of more susceptible lines in the later NC generations.

The total incidences in the uninjected mice of the MCN and MNC groups also differed significantly.[31] As previously mentioned (Miller and Pybus, 1954), although the pure strain parents of the hybrids came from several different inbred lines, the bulk of the MCN and MNC mice were the offspring of reciprocal crosses between NBT Line C and CBA Line P. Since all the MCN and MNC mice of the first 4 generations were injected, there were no uninjected animals of these 2 groups available for comparison until $F_5$, by which time lines of different susceptibilities could have segregated. There was considerable fluctuations in incidence from generation to generation (Tables VI and X) and the actual numbers of tumours in each generation, especially in the uninjected MCN group, were often too small for individual analysis. The uninjected MCN and MNC mice were better controls, genetically, for the later generations of the injected groups than were the CN and NC hybrids.

B. *Induced tumours.*

The data for induced lung tumour incidences in all generations of the reciprocal hybrids are given in Tables XIV and XV. For the sake of comparison between the whole groups, the figures are based on the numbers of mice alive at the time of the earliest tumour in each whole group (6 months for MCN, 5 months for MNC), so that in these tables the $F_1$ and $F_2$ incidences are less than the corresponding figures in Table III, where (for comparison between the individual generations) the incidences were based on the numbers alive at 8 and 7 months respectively in the MCN and MNC $F_1$'s, and at 7 and 6 months in the $F_2$'s.

In all 10 generations of each group of injected hybrids the tumour incidences remained high, but with wide fluctuations. In the MCN group (Table XIV) the total incidences were 35·9 per cent in 468 females and 24·3 per cent in 445 males, a significant difference.[32] In the MNC hybrids (Table XV) the incidences were 34·5 per cent in 643 females and 22·2 per cent in 744 males, also a significant difference.[33] The differences between the hybrids were not significant for either sex.

TABLE XIV.—*Incidence of Induced Lung Tumours in Mice of each Generation of MCN Hybrids, Living to* 6 *Months and Over.*

| Generation. | Sex. | Number of mice. Total in experiment. | Surviving to tumour age. | Tumorous mice. Number. | Percentage. |
|---|---|---|---|---|---|
| $F_1$ | Females | 47 | 44 | 18 | 40·9 |
| | Males | 50 | 49 | 15 | 30·6 |
| $F_2$ | Females | 134 | 111 | 45 | 40·5 |
| | Males | 120 | 99 | 34 | 34·3 |
| $F_3$ | Females | 45 | 44 | 26 | 59·1 |
| | Males | 46 | 45 | 14 | 31·1 |
| $F_4$ | Females | 59 | 54 | 17 | 31·5 |
| | Males | 58 | 47 | 10 | 21·3 |
| $F_5$ | Females | 58 | 52 | 9 | 17·3 |
| | Males | 66 | 56 | 10 | 17·9 |
| $F_6$ | Females | 12 | 11 | 6 | 54·5 |
| | Males | 13 | 6 | 1 | 16·7 |
| $F_7$ | Females | 25 | 23 | 5 | 21·7 |
| | Males | 25 | 20 | 2 | 10·0 |
| $F_8$ | Females | 53 | 44 | 17 | 38·6 |
| | Males | 70 | 56 | 9 | 16·1 |
| $F_9$ | Females | 39 | 35 | 11 | 31·4 |
| | Males | 44 | 31 | 5 | 16·1 |
| $F_{10}$ | Females | 66 | 50 | 14 | 28·0 |
| | Males | 50 | 36 | 8 | 22·2 |
| Total | Females | 538 | 468 | 168 | 35·9 |
| | Males | 542 | 445 | 108 | 24·3 |

In each of the first 2 generations, the tumour incidences in males and females did not differ significantly whether based on the earlier tumour ages in Tables XIV and XV or on the later ages in Table III. The mice lived longer in the earlier than in the later generations. It was proved on further analysis that the sex-difference in each group was due to a greater proportion of males than females dying young. When the figures were corrected, in exactly the same way as shown in Table XII for the NC mice, to what they would have been if the males

TABLE XV.—*Incidence of Induced Lung Tumours in Mice of each Generation of MNC Hybrids Living to 5 Months and Over.*

| Generation. | Sex. | Number of mice. Total in experiment. | Number of mice. Surviving to tumour age. | Tumorous mice. Number. | Tumorous mice. Percentage. |
|---|---|---|---|---|---|
| $F_1$ | Females | 51 | 48 | 15 | 31·3 |
| | Males | 54 | 51 | 13 | 25·5 |
| $F_2$ | Females | 122 | 121 | 39 | 32·2 |
| | Males | 125 | 125 | 40 | 32·0 |
| $F_3$ | Females | 33 | 33 | 11 | 33·3 |
| | Males | 36 | 36 | 6 | 16·7 |
| $F_4$ | Females | 107 | 105 | 39 | 37·1 |
| | Males | 110 | 104 | 27 | 26·0 |
| $F_5$ | Females | 60 | 58 | 20 | 34·5 |
| | Males | 88 | 82 | 17 | 20·7 |
| $F_6$ | Females | 49 | 46 | 11 | 23·9 |
| | Males | 51 | 48 | 10 | 20·8 |
| $F_7$ | Females | 84 | 83 | 23 | 27·7 |
| | Males | 96 | 93 | 13 | 14·0 |
| $F_8$ | Females | 31 | 29 | 15 | 51·7 |
| | Males | 50 | 50 | 11 | 22·0 |
| $F_9$ | Females | 62 | 57 | 22 | 38·6 |
| | Males | 79 | 76 | 15 | 19·7 |
| $F_{10}$ | Females | 70 | 63 | 27 | 42·9 |
| | Males | 81 | 79 | 13 | 16·5 |
| Total | Females | 669 | 643 | 222 | 34·5 |
| | Males | 770 | 744 | 165 | 22·2 |

had lived to the same ages as the females, it was found that in the MCN group there would have been 161 tumour males or 36·2 per cent, and in the MNC group there would have been 252 tumour males or 33·9 per cent. These figures were in agreement with the incidences in the females in each group.

As shown in Table XVI, tumours were found later in the females than in the males of both groups, the sex-difference being significant. The average ages at death of the non-tumour mice, all of which lived to the lowest tumour age for their group, were very much less than the average ages at death of the tumour mice. There was no sex-difference in age at death of non-tumour mice. A high proportion of non-tumour mice of both sexes died before mean tumour age.

In both groups the incidence of induced tumours was much greater than that of spontaneous tumours and the tumour age was significantly lowered. The lower age may have been due to injected mice coming to autopsy at an earlier age, tumours therefore being seen earlier, or the tumours may have actually been induced earlier.

C. *Multiple nodules.*

All the tumour-bearing mice were classified into 2 groups, those with solitary tumours and those with multiple (two or more) nodules, as observed with the

TABLE XVI.—*Average Ages at Death of Tumour and Non-tumour MCN and MCN Hybrids.*

| | Tumour females. | | | Tumour males. | | | Non-tumour females. | | | Non-tumour males. | | | | |
|---|---|---|---|---|---|---|---|---|---|---|---|---|---|---|
| Strain. | Number. | Average age at death (months). | σ. | Number. | Average age at death (months). | σ. | Number. | Average age at death (months). | σ. | Number. | Average age at death (months). | σ. | Earliest tumour (months). | Latest tumour (months). |
| MCN . | 168 | 15·6 | 4·8 | 108 | 14·1 | 4·7 | 300 | 8·7 | 4·7 | 337 | 8·0 | 3·7 | 6 | 28 |
| MNC . | 222 | 15·6 | 5·6 | 165 | 13·7 | 5·3 | 447 | 7·8 | 3·0 | 579 | 8·0 | 3·4 | 5 | 32 |

naked-eye. The post-mortem records did not always state the exact number of nodules, unless solitary.

In the control groups, Generations 1 to 12 (Table IV), the majority of mice had single tumours, but there were some with 2 and a very few with a greater number. In the injected groups, Generations 1 to 10 (Table IV), the proportions were reversed, the majority of mice having 2 or more nodules and many having "multiple" nodules.

There was no sex-difference in any of the groups except MNC, where 34·2 per cent of tumour females and 47·9 per cent of tumour males had single nodules, the difference of 13·7 per cent being significant.[34] Heston and Deringer (1952) found a greater number of multiple nodules in female guinea pigs.

The differences in numbers of nodules between the reciprocal hybrids were not significant as regards either spontaneous or induced tumours. There was a significant difference of 15·4 per cent between the NC and the uninjected MNC groups,[35] the latter having the greater proportion of solitary tumours; it has been shown above that the latter group is more resistant. But the uninjected MNC mice also had a significantly greater proportion of solitary nodules (91·7 per cent) than the uninjected MCN mice (77·5 per cent),[36] although the latter were believed to be the more resistant group.

### DISCUSSION.

### 1. *The anomalous $F_1$ result.*

The analysis of lung tumour incidence in the $F_1$ reciprocal hybrids showed no difference between spontaneous and induced tumours. This was contrary to all previous work in this field, which has established beyond doubt that the incidence of lung tumours is increased in mice treated with carcinogens, whether adminstered by painting, by subcutaneous or by intravenous injection (Lynch, 1927; Andervont, 1937*a*, 1939; Shimkin, 1940*a*, 1940*b*; Andervont and Shimkin, 1940; Heston, 1940).

Although a number of different matings between the pure strains had been made, half the mice of each $F_1$ litter were injected, so that the contribution of the various matings to the injected and uninjected groups were similar, and there could be no question of the control $F_1$ animals differing from the injected in susceptibility.

It was then discovered that, by what is now realised to have been an error of judgment, but partly to ensure uniformity of environment for treated and untreated mice and also for the compelling reasons of available space and labour, in many instances (but by no means in all) the injected and uninjected $F_1$ mice of the same litter were kept in the same box either throughout their lives or until transferred to the breeding boxes. This occurred only with $F_1$; in all the other generations the injected and uninjected mice were kept apart.

It is possible that some of the carcinogen, oozing out on to the coats of the injected animals as it is liable to do after subcutaneous injection, was transferred to the uninjected either by skin contact or by licking. The amount may have been small in proportion to that injected; no skin tumours were found in the controls; the average lung tumour age was much greater in the $F_1$ controls (possibly because the latter lived much longer) than in the treated animals; the majority of the control tumorous mice had solitary lung nodules in contrast to the high proportion of multiple nodules in the treated mice. But quite small amounts of carcinogen are known to produce lung tumours by intravenous injection in Strain A mice, and the weaker the dose, the fewer nodules per mouse (Shimkin, 1940*b*; Shimkin and McClelland, 1949). Andervont and Dunn (1953) obtained a few lung tumours in $DBA_f/2$ mice by oral administration of methylcholanthrene. Bagshaw and Strong (1950) did not believe that such quantities as might be ingested in this way could induce tumours of the forestomach, but Andervont and Dunn (1953) discovered that mammary tumours could be induced by oral administration of methylcholanthrene after they had suggested (Andervont and Dunn, 1950) that the licking habits of mice might be responsible for some of their earlier results.

To test the possibility that such licking habits might be the cause of the high $F_1$ incidences in the present work, the incidences of tumours of the forestomach (both papillomata and epitheliomata) were analysed, but these showed no comparable effect. In the untreated $F_1$ reciprocal hybrids the incidence of these tumours was about 14 per cent; in the treated $F_1$ hybrids the incidence was about 60 per cent and the tumours were seen from 7 to 8 months earlier, differences which were significant and implied a definite action by the carcinogen in the treated mice. Gross epitheliomata were found much more frequently in the treated animals. It is intended to make these tumours the subject of a subsequent communication.

Different tissues are known to have different susceptibilities to the same carcinogen (Andervont and Shimkin, 1940; Rask-Nielsen, 1950) and a dose which can induce lung tumours might be ineffective in the case of the forestomach, although it might be thought that oral administration would be more likely to affect the stomach than the lungs. The figures for mammary tumour incidence in the present material are now being reconsidered in the light of the present discovery and of the results of Andervont and Dunn (1953), and may likewise show the effect of the $F_1$ contamination, although a first analysis suggested other causes for the appearance of these tumours.

The conclusion is, therefore, that while it is not certain that any contamination took place in the $F_1$ mice, or that a sufficiently potent quantity of the carcinogen was transferred in the event of such contamination, the possibility of the occurrence does provide an explanation of the anomalous result and must be borne in mind both in the present case and in the analysis of other types of remote neoplasms in the reciprocal hybrids.

2. *Comparison of reciprocal hybrids.*

If the high incidences of lung tumours in " untreated " $F_1$ mice were due to the ingestion of small amounts of methylcholanthrene, it is not possible to state definitely whether the reciprocal $F_1$ hybrids were alike in susceptibility to spontaneous lung tumours. It seems probable that they were, since previous work has shown that susceptibility to induced tumours parallels that to spontaneous tumours (Andervont, 1937*a*; Shimkin, 1940*a*). Heston (1942*a*) found no difference between reciprocal $F_1$ hybrids (between a susceptible and a resistant strain) as regards induced tumours, and presumably none in regard to spontaneous tumours (Heston, 1942*b*) since both lots of hybrids were combined in one group.

In the present case, the incidences of spontaneous tumours in $F_2$ were less than in $F_1$ in both groups of hybrids. The incidences in later generations diverged, CN mice becoming less susceptible, while the incidence in NC remained fairly high. Heston (1942*a*) and Andervont (1937*b*) both reported $F_2$ to be less susceptible than $F_1$ to induced tumours ; this was not observed in the present work, where the two generations showed no significant difference.

The incidence of spontaneous tumours in the early generations of hybrids was higher than in the parent strains, even excluding $F_1$. Andervont (1940) observed the same phenomenon with regard to induced tumours, but the present material did not show it for induced tumours. Although each of the parent strains had a relatively low susceptibility to spontaneous tumours, the incidence of induced tumours in CBA mice was higher than in the reciprocal $F_1$ hybrids but not significantly so. The reciprocal hybrids were equally susceptible to induced tumours ; this was shown both by the numbers of tumorous mice and by the percentages with multiple lung nodules. Heston and Deringer (1949) found the data on incidence less striking than the difference in number of nodules.

The fact that, in spite of the suspected contamination of $F_1$, the tumour incidences fell in later generations of CN hybrids and also in the uninjected MCN and MNC mice (descendants of treated animals) confirms the opinion that the effect of the carcinogen was confined to those mice which were exposed to it and that there was no continuing effect on their untreated offspring. Strong (1943) obtained a new line of mice characterised by a high incidence of lung carcinoma after hybridisation (and methylcholanthrene injection in earlier generations) and believed this to be due to a continuing inheritable effect of the carcinogen.

No real sex-difference was observed in the incidence of either spontaneous tumours (as noted also by Shimkin, 1940*b* ; and by Heston and Deringer, 1947) or induced tumours (as found also by Andervont, Grady and Edwards, 1942 ; Heston, 1942*a* ; Strong, 1943 ; and many others). Apparent sex-differences were found to be due to early deaths of the males. For the same reason, the average tumour age was usually earlier for males than females (as noted also by Bittner, 1936).

### SUMMARY.

1. The incidence of spontaneous lung tumours was investigated in the inbred strains of mice, CBA and NBT, and in their reciprocal hybrids, and compared with the incidence of lung tumours induced in the same material by subcutaneous injection of methylcholanthrene.

2. The CBA mice were more susceptible to both spontaneous and induced lung tumours than the NBT mice. There was no difference between the reciprocal hybrids in susceptibility to induced tumours or, in the early generations, to spontaneous tumours. Twelve generations of inbreeding caused genetical divergence in the 2 groups of hybrids, the CN mice becoming less susceptible to spontaneous lung tumours and the NC mice retaining a moderately high susceptibility. The reciprocal hybrids, in the early generations, were more susceptible than the parent strains to spontaneous tumours.

3. The incidence of lung tumours and the proportion of tumorous mice bearing multiple lung nodules were greatly increased by injection of 1·0 mg. methylcholanthrene. This dose was sufficiently high to obliterate the genetical divergence of the 2 groups of hybrids.

4. The mean tumour age for induced tumours was much less than that for spontaneous tumours. This may have been due to the earlier deaths of treated animals, whereby the tumours were seen earlier, but may also have been due to an acceleration of the process leading to neoplasia.

5. There was no real sex-difference in the incidences of spontaneous or of induced lung tumours. Both types of tumours were found later in females owing to the longer life of the latter.

6. There was no indication of a persistence of the effect of methylcholanthrene in raising the lung tumour incidence. Uninjected generations regained the low incidence of spontaneous tumours characteristic of their group.

7. The effect of a possible contamination of the untreated $F_1$ hybrids with slight amounts of methylcholanthrene is discussed with regard both to lung tumours and other types of remote neoplasms. The possibility of this contamination was discovered when the tumour incidences in injected and uninjected $F_1$ mice were found to be the same.

We wish to thank Dr. U. Philip for her interest and advice in the preparation of this paper.

This investigation was carried out with the aid of a research grant from the North of England Branch of the British Empire Cancer Campaign.

### REFERENCES.

Andervont, H. B.—(1937*a*) *Publ. Hlth. Rep., Wash.*, **52**, 212.—(1937*b*) *Ibid.*, **52**, 304.—(1939) *Ibid.*, **54**, 1512.—(1940) *J. nat. Cancer Inst.*, **1**, 135.

*Idem* and Dunn, T. B.—(1950) *Ibid.*, **10**, 895.—(1953) *Ibid.*, **14**, 329.

*Idem*, Grady, H. G., and Edwards, J. E.—(1942) *Ibid.*, **3**, 131.

*Idem* and Shimkin, M. B.—(1940) *Ibid.*, **1**, 225.

Bagshaw, M. A., and Strong, L. C.—(1950) *Ibid.*, **11**, 141.

Bittner, J. J.—(1936) *Amer. J. Cancer*, **27**, 519.

Heston, W. E.—(1940) *J. nat. Cancer Inst.*, **1**, 105.—(1942*a*) *Ibid.*, **3**, 69.—(1942*b*) *Ibid.*, **3**, 79.

*Idem* and Deringer, M. K.—(1947) *Ibid.*, **7**, 463.—(1949) *Ibid.*, **10**, 119.—(1952) *Ibid.*, **13**, 705.

Lynch, C. J.—(1927) *J. exp. Med.*, **46**, 917.—(1940) *Proc. Soc. exp. Biol. N.Y.*, **43**, 186.

Miller, E. W., and Pybus, F. C.—(1952) *Ann. Rep. Brit. Emp. Cancer Campgn.*, **30**, 205.—(1954) *Brit. J. Cancer*, **8**, 163.

Rask-Nielsen, R.—(1950) *Ibid.*, **4**, 108.

Shimkin, M. B.—(1940*a*) *Arch. Path.*, **29**, 229.—(1940*b*) *Ibid.*, **29**, 239.

*Idem* and McClelland, J. N.—(1949) *J. nat. Cancer Inst.*, **10**, 597.

Strong, L. C.—(1936) *Brit. J. exp. Path.*, **17**, 60.—(1940) *Amer. J. Cancer*, **39**, 347.—(1943) *Arch. Path.*, **36**, 58.—(1945) *J. nat. Cancer Inst.*, **5**, 339.

### NOTES.

1 $\chi^2 = 2{\cdot}9$, $P < 0{\cdot}05$ in CBA. $\chi^2 = 0{\cdot}3$, $P > 0{\cdot}5$ in NBT.

2 $\chi^2 = 10{\cdot}0$, $P < 0{\cdot}01$.

3 $2 \times \sigma d = 4{\cdot}06$ ; $d = 4{\cdot}125$ in NBT. $2 \times \sigma d = 2{\cdot}104$ ; $d = 3{\cdot}862$ in CBA.

4 $\chi^2 = 1{\cdot}4$, $P > 0{\cdot}2$.

5 $\chi^2 = 0{\cdot}2$, $P > 0{\cdot}5$.

6 $\chi^2 = 17{\cdot}7$, $P < 0{\cdot}01$.

7 $2 \times \sigma d = 2{\cdot}8$, $d = 3{\cdot}3$.

8 Difference between percentages = 49·2, 2 × standard error = 34·7.

9 $\chi^2 > 9{\cdot}7$, $P < 0{\cdot}01$.

10 $2\sigma d = 3{\cdot}8$, $d = 6{\cdot}3$ in M/CBA. $2\sigma d = 4{\cdot}8$, $d = 2{\cdot}0$ in M/NBT.

11 All CN *v.* CN $F_1$. ♀♀ : $d = 1{\cdot}9$, $2\sigma d = 2{\cdot}4$ ; ♂♂ : $d = 1{\cdot}9$, $2\sigma d$ 1·5.

All NC *v.* NC $F_1$. ♀♀ : $d = 3{\cdot}3$, $2\sigma d = 2{\cdot}3$ ; ♂♂ : $d = 1{\cdot}0$, $2\sigma d = 1{\cdot}8$.

12 CN $F_1$ ♀♀ *v.* ♂♂ : $d = 2{\cdot}7$, $2\sigma d = 2{\cdot}534$.

NC $F_1$ ♀♀ *v.* ♂♂ : $d = 5{\cdot}5$, $2\sigma d = 2{\cdot}586$.

13 CN ♀♀ *v.* NC ♀♀ : $d = 0{\cdot}1$, $2\sigma d = 3{\cdot}12$.

CN ♂♂ *v.* NC ♂♂ : $d = 2{\cdot}7$, $2\sigma d = 1{\cdot}829$

14 $\chi^2 = 9{\cdot}8$, $P < 0{\cdot}01$.

15 $\chi^2 = 5{\cdot}375$, $P < 0{\cdot}05$ and $> 0{\cdot}02$.

16 CN $F_1$ ♂♂ *v.* CN $F_2$ ♂♂ : $d = 2{\cdot}1$, $2\sigma d = 1{\cdot}7$.

17 All MCN *v.* MCN $F_1$ (males) $d = 5{\cdot}9$, $2\sigma d = 2{\cdot}4$.

All MNC *v.* MNC $F_1$ (males) $d = 6{\cdot}9$, $2\sigma d = 3{\cdot}3$.

18 MCN $F_2$ *v.* CN $F_2$. $\chi^2 = 15$, $P < 0{\cdot}01$.

MNC $F_2$ *v.* NC $F_2$. $\chi^2 = 12$, $P < 0{\cdot}01$.

19 MCN $F_1$ *v.* $F_2$ (males) $d = 6{\cdot}0$, $2\sigma d = 2{\cdot}7$.

MNC $F_1$ *v.* $F_2$ (males) $d = 4{\cdot}9$, $2\sigma d = 3{\cdot}8$.

20 CN $F_1$ *v.* MCN $F_1$. $\chi^2 > 8$, $P < 0{\cdot}01$. NC $F_1$ *v.* MNC $F_1$. $\chi^2 > 10$, $P < 0{\cdot}01$.

CN $F_2$ *v.* MCN $F_2$. $\chi^2 > 29$, $P < 0{\cdot}01$. NC $F_2$ *v.* MNC $F_2$. $\chi^2 > 9$, $P < 0{\cdot}01$.

21 $\chi^2 > 26$, $P < 0{\cdot}01$.

$\chi^2 > 14$, $P < 0{\cdot}01$ ($F_2$–$F_{12}$).

22 $d = 5{\cdot}9$, $2\sigma d = 0{\cdot}74$ (CN ♀♀ *v.* ♂♂).

23 $d = 1{\cdot}3$, $2\sigma d = 0{\cdot}56$ (uninjected MCN ♀♀ *v.* ♂♂).

24 CN ♀♀ *v.* MCN ♀♀ : $d = 5{\cdot}7$, $2\sigma d = 0{\cdot}69$.

CN♂♂ v. MCN ♂♂ $d = 1{\cdot}1$, $2\sigma d = 0{\cdot}62$.

25 $\chi^2 > 10{\cdot}8$, $P < 0{\cdot}01$.

$\chi^2 > 14{\cdot}8$, $P < 0{\cdot}01$ ($F_2$–$F_{12}$).

26 $\chi^2 > 9{\cdot}4$, $P < 0{\cdot}01$.

27 $d = 5{\cdot}9$, $2\sigma d = 0{\cdot}74$.

28 ♀♀ : $d = 1{\cdot}8$, $2\sigma d = 0{\cdot}8$. ♂♂ : $d = 5{\cdot}0$, $2\sigma d = 0{\cdot}6$.

29 ♀♀ : $d = 1{\cdot}6$, $2\sigma d = 1{\cdot}4$ ; ♂♂ : $d = 4{\cdot}7$, $2\sigma d = 1{\cdot}8$.

30 $\chi^2 > 12$, $P < 0{\cdot}01$.

31 $\chi^2 > 8{\cdot}9$, $P < 0{\cdot}01$.

32 $\chi^2 > 10$, $P < 0{\cdot}01$.

33 $\chi^2 > 16$, $P < 0{\cdot}01$.

34 $d = 13{\cdot}7$, 2 × standard error = 9·8.

35 $d = 15{\cdot}4$, 2 × standard error = 7·7.

36 $d = 14{\cdot}2$, 2 × standard error = 13·9.


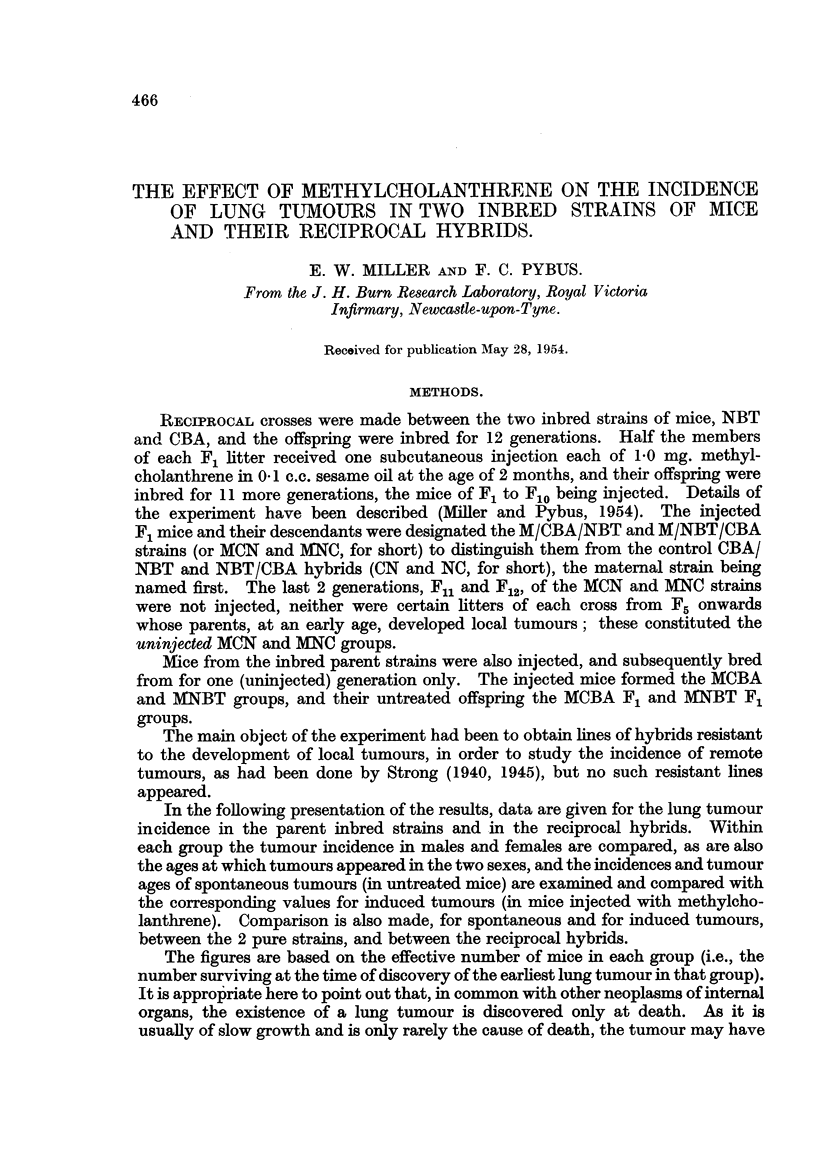

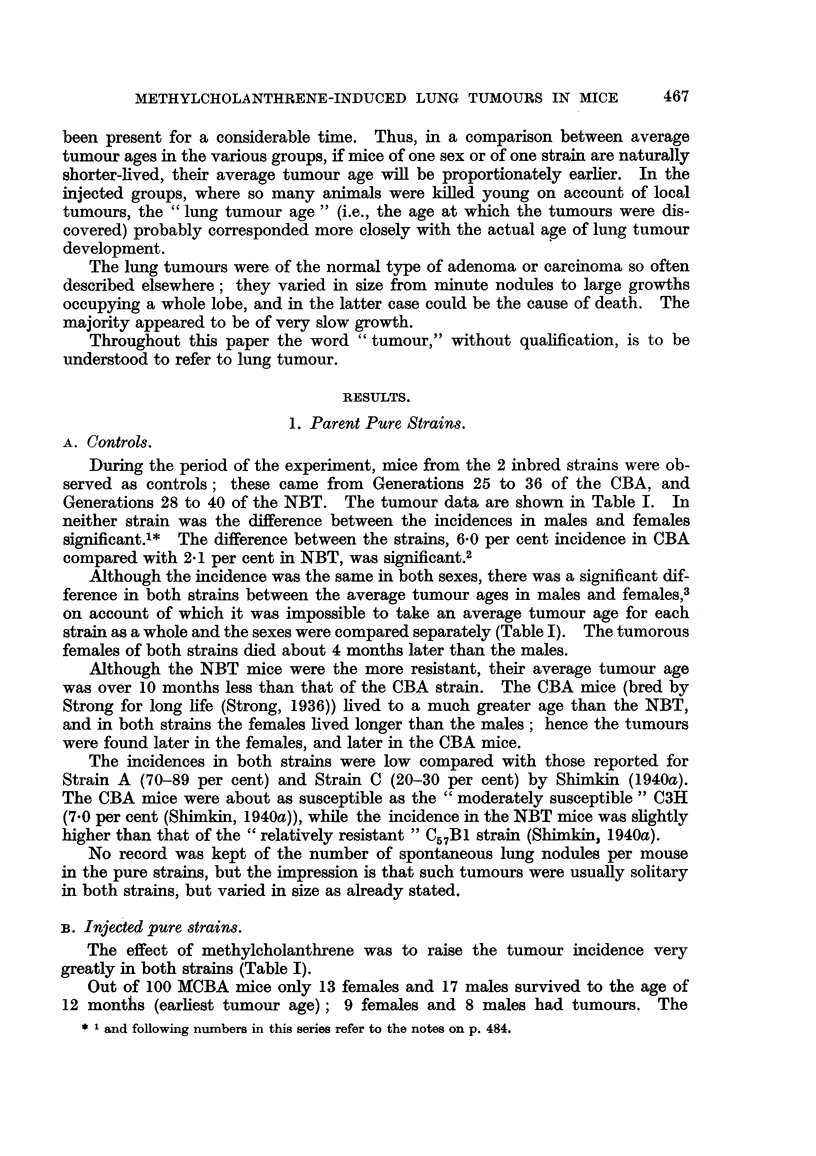

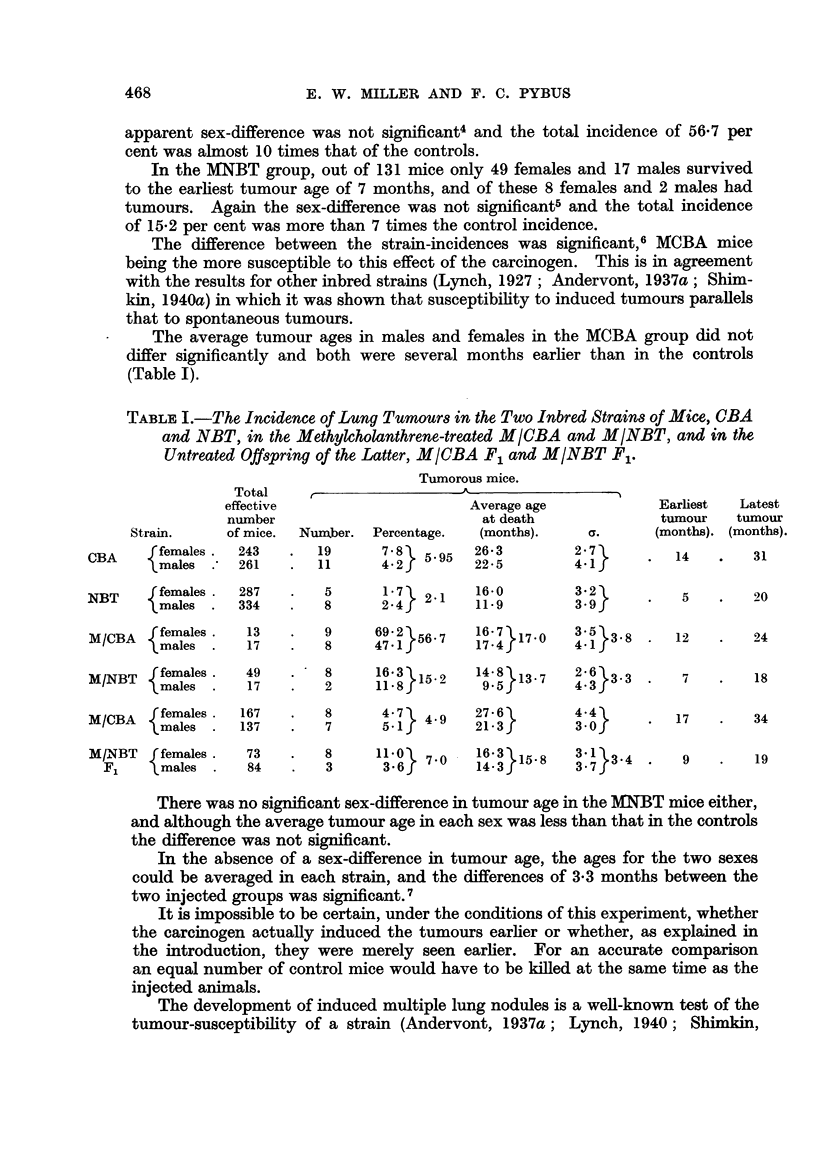

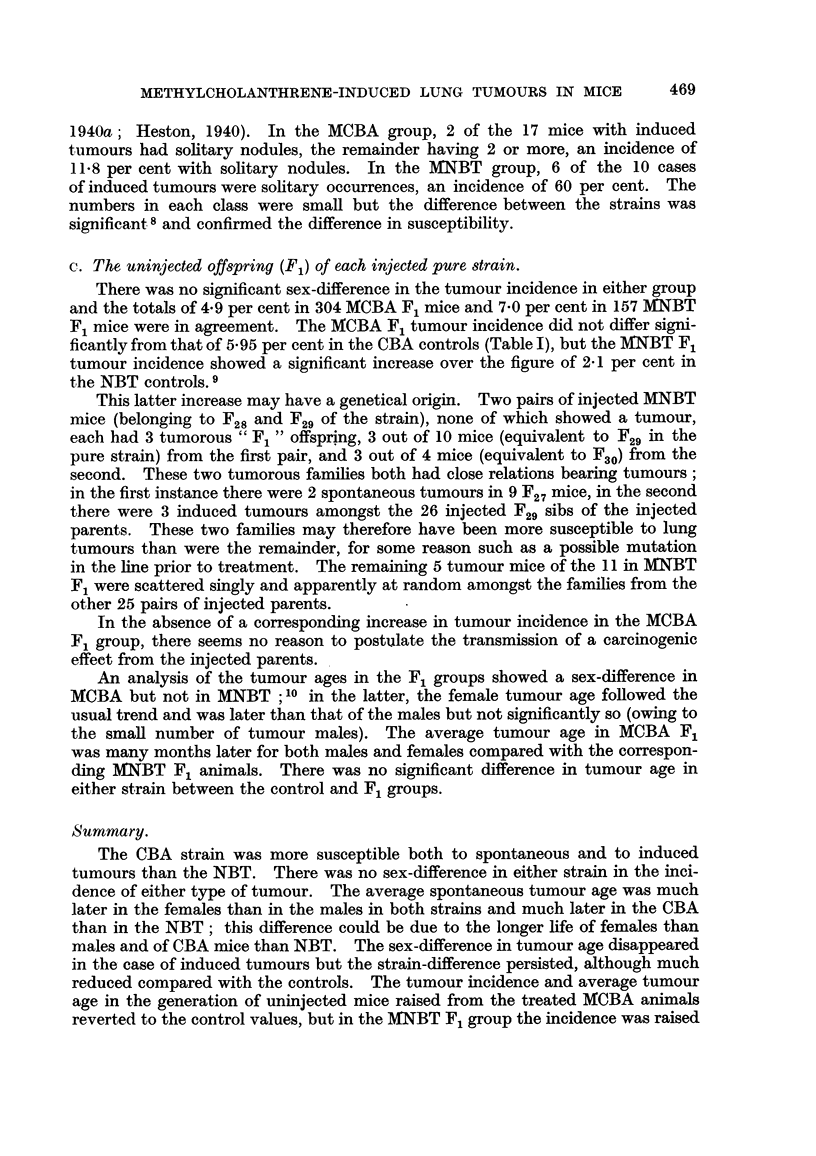

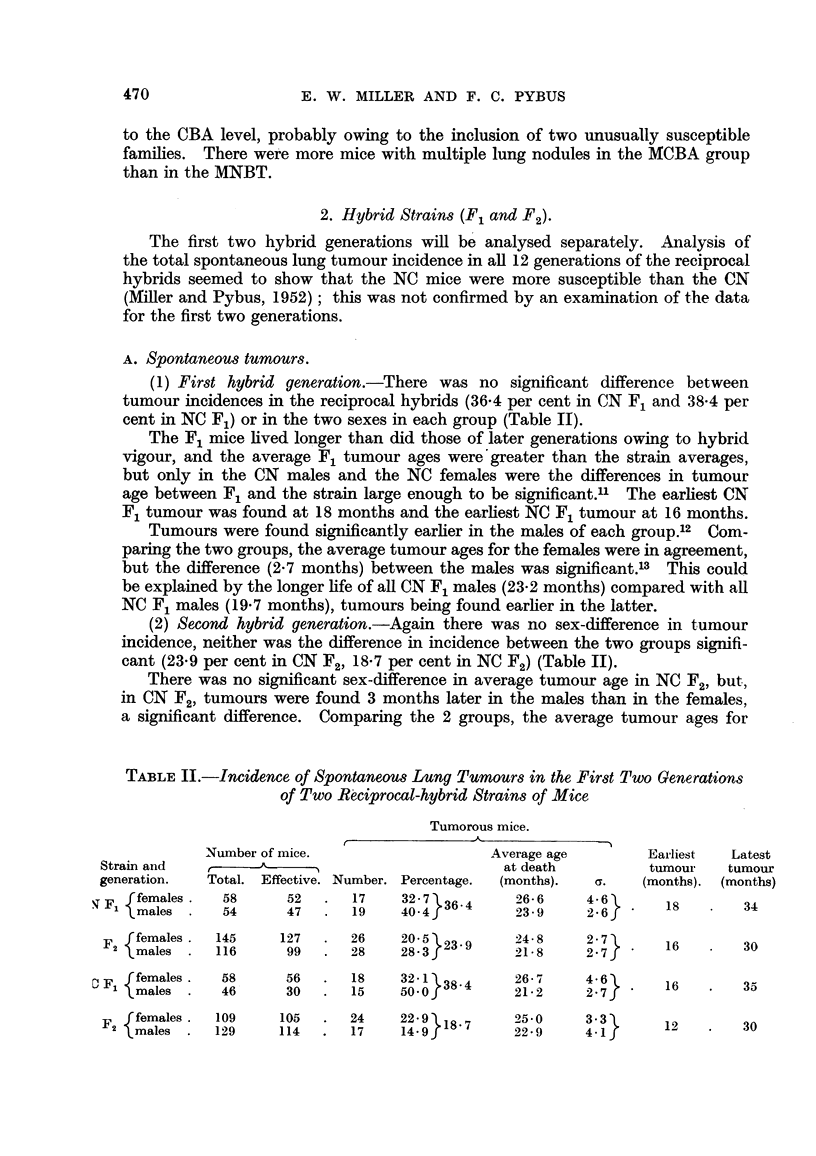

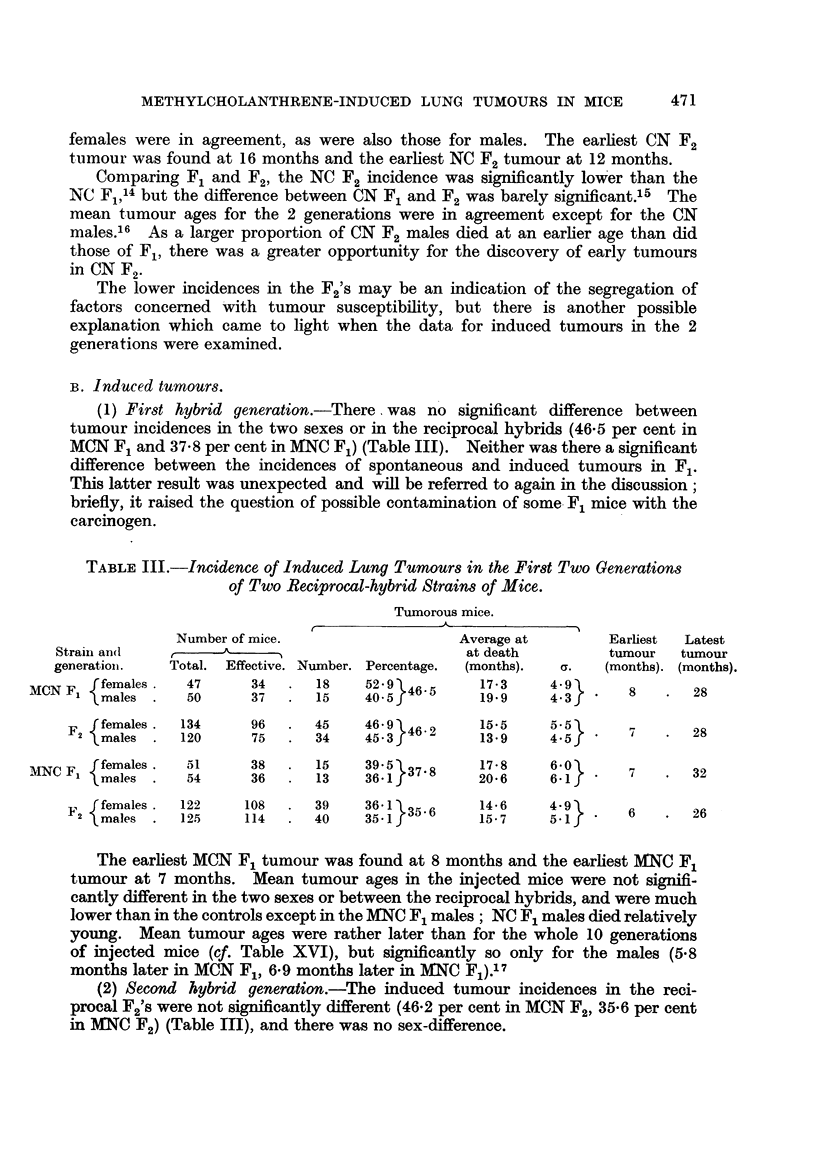

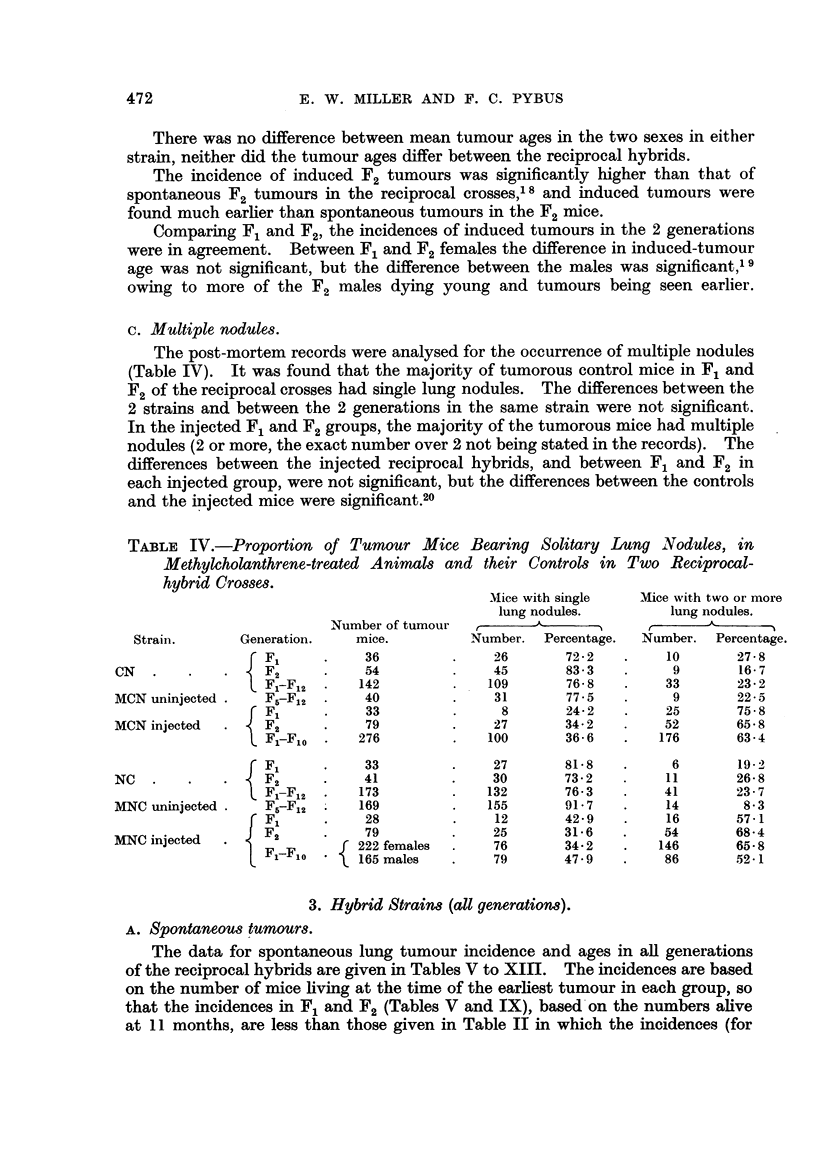

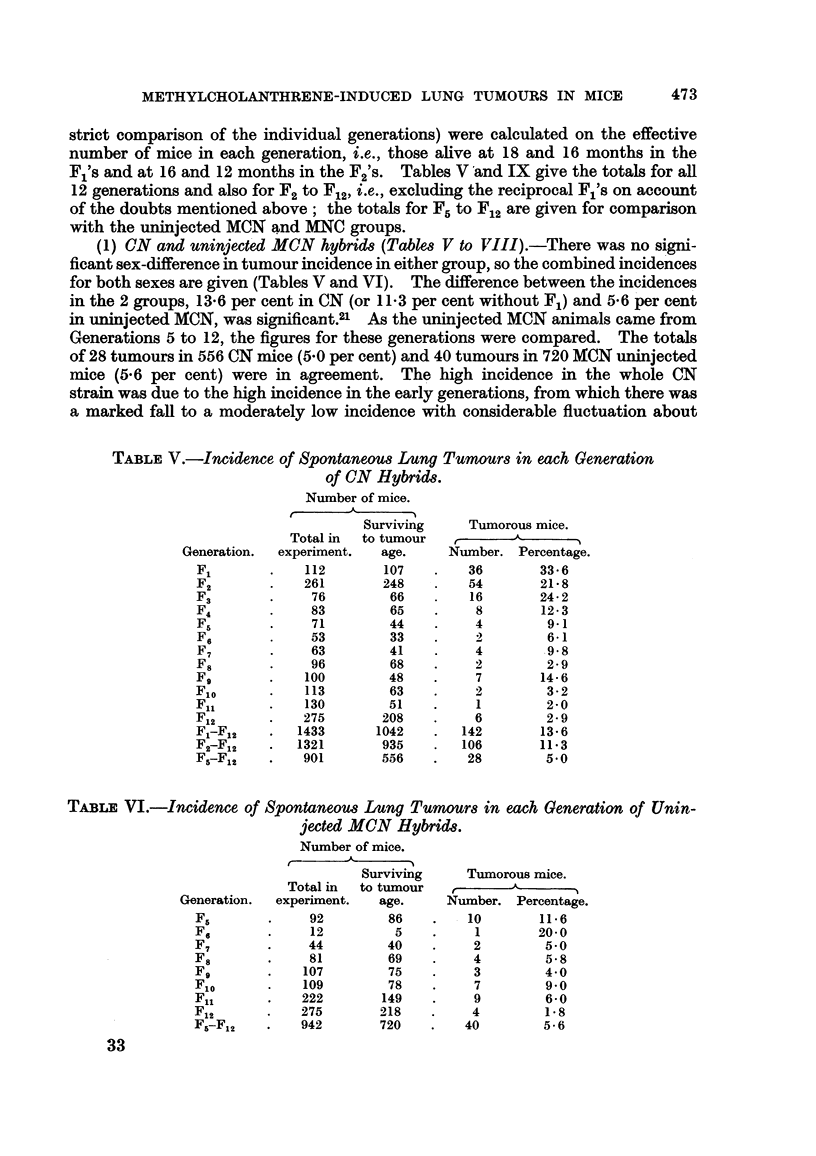

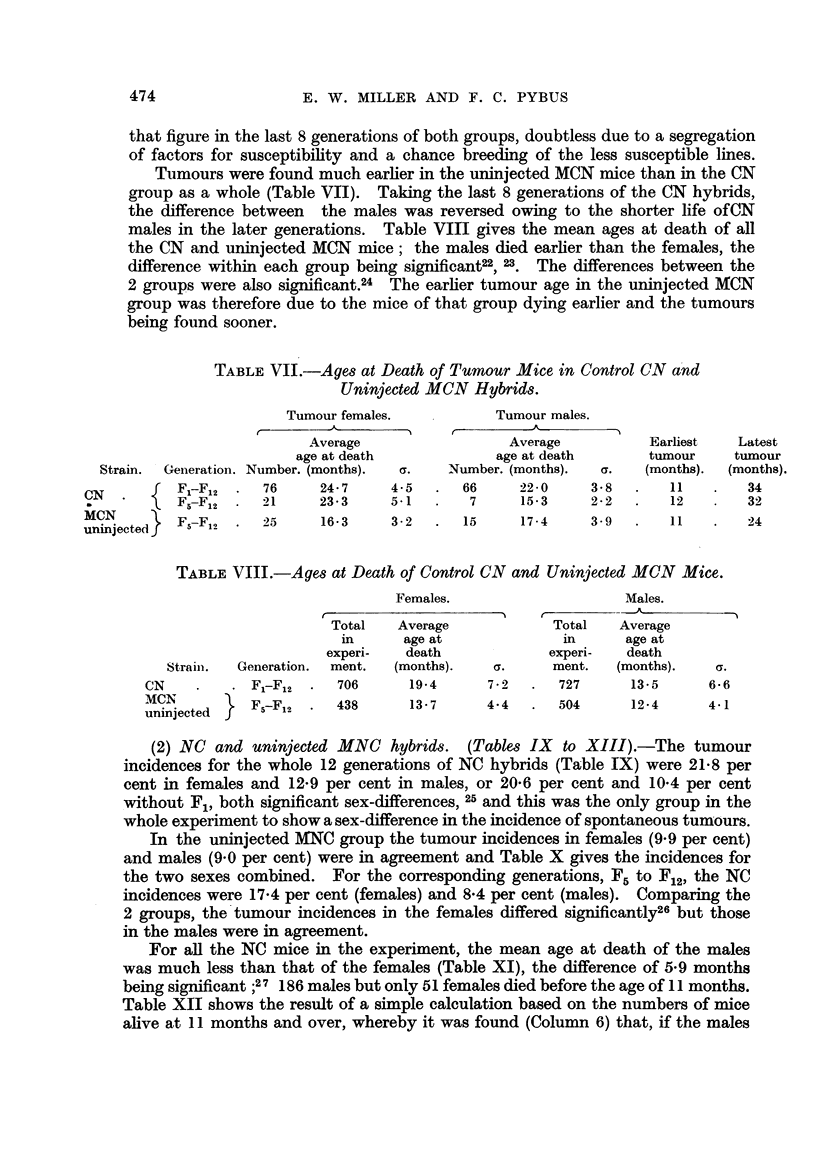

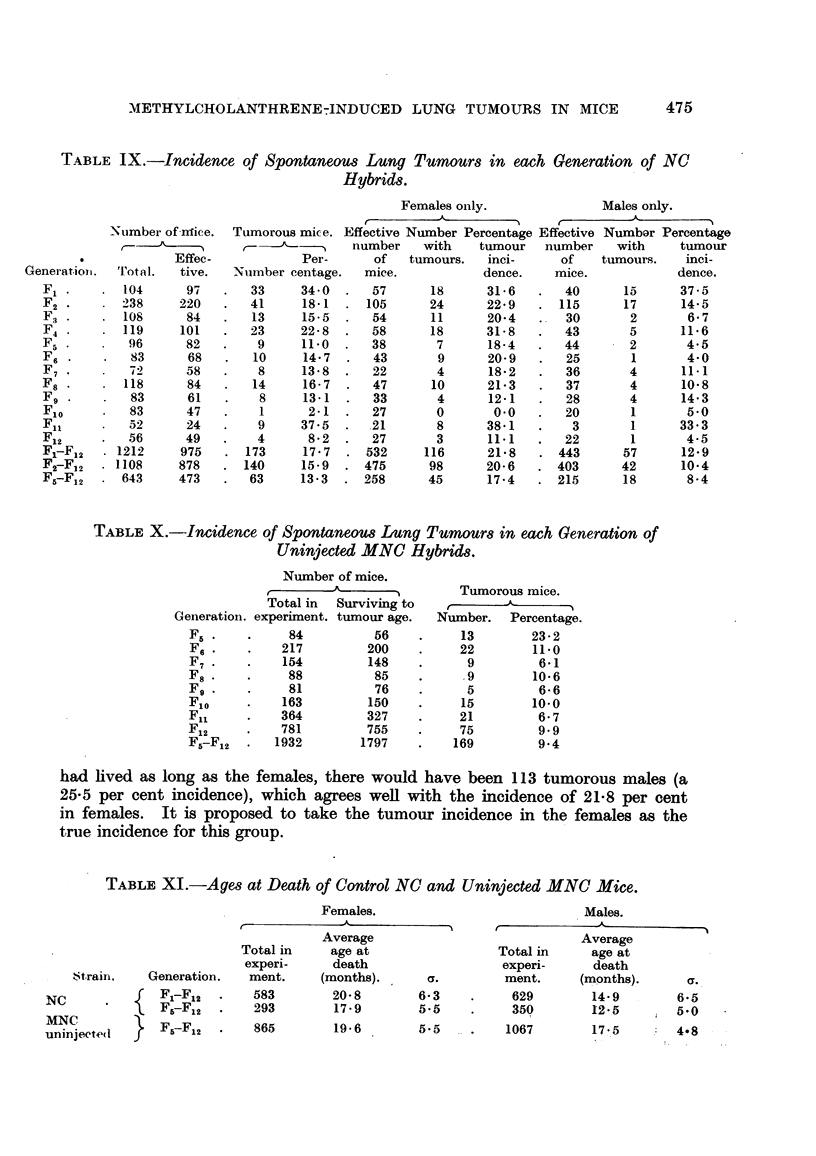

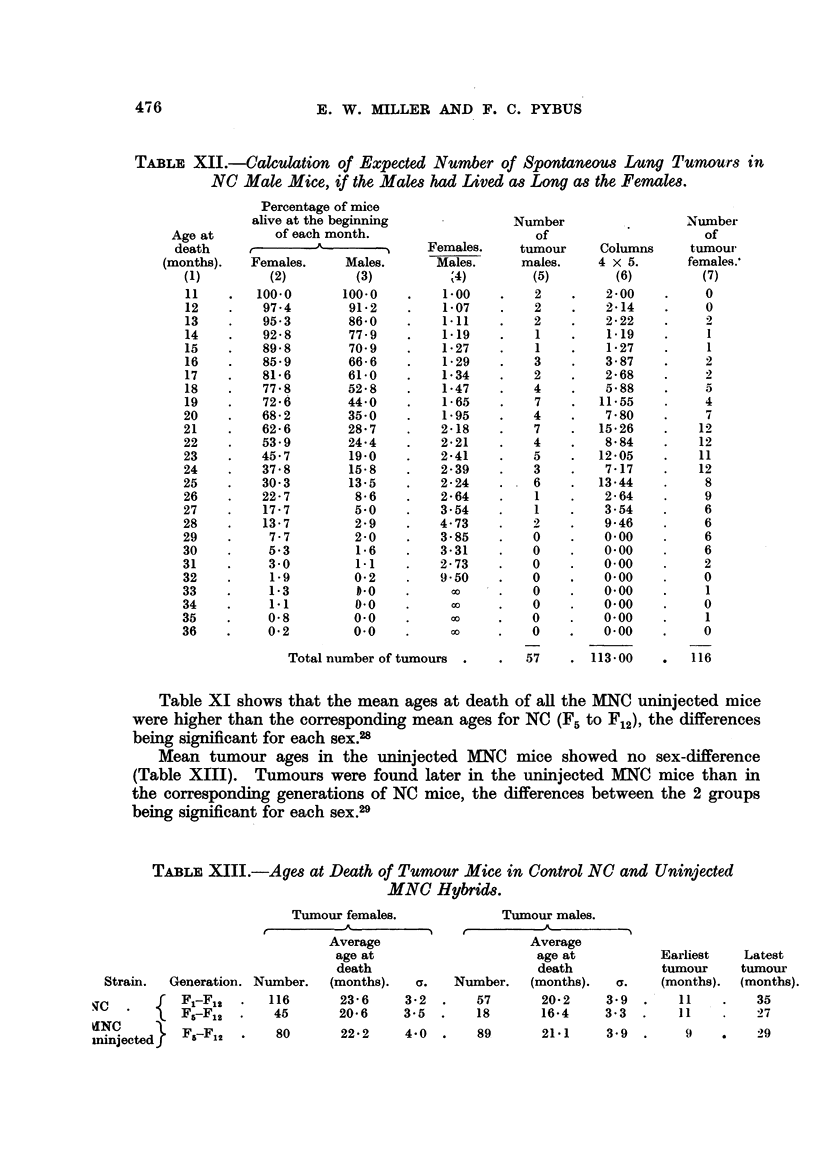

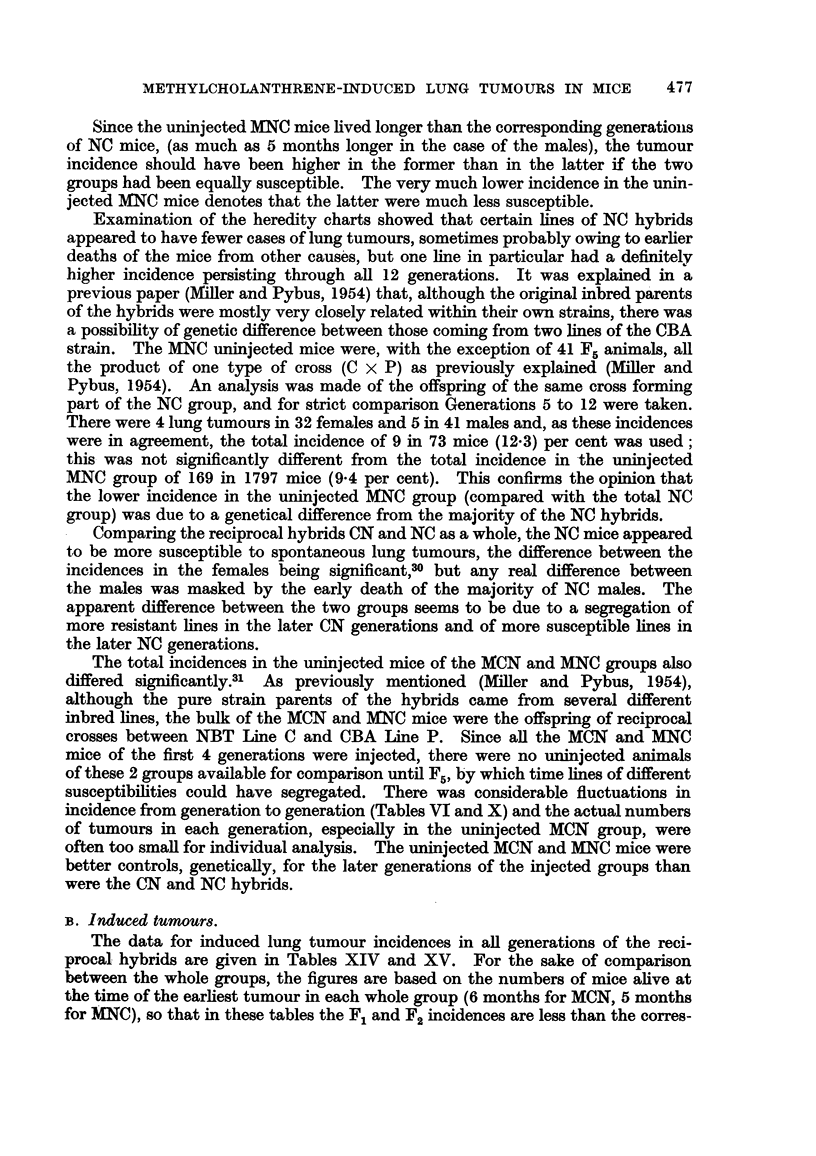

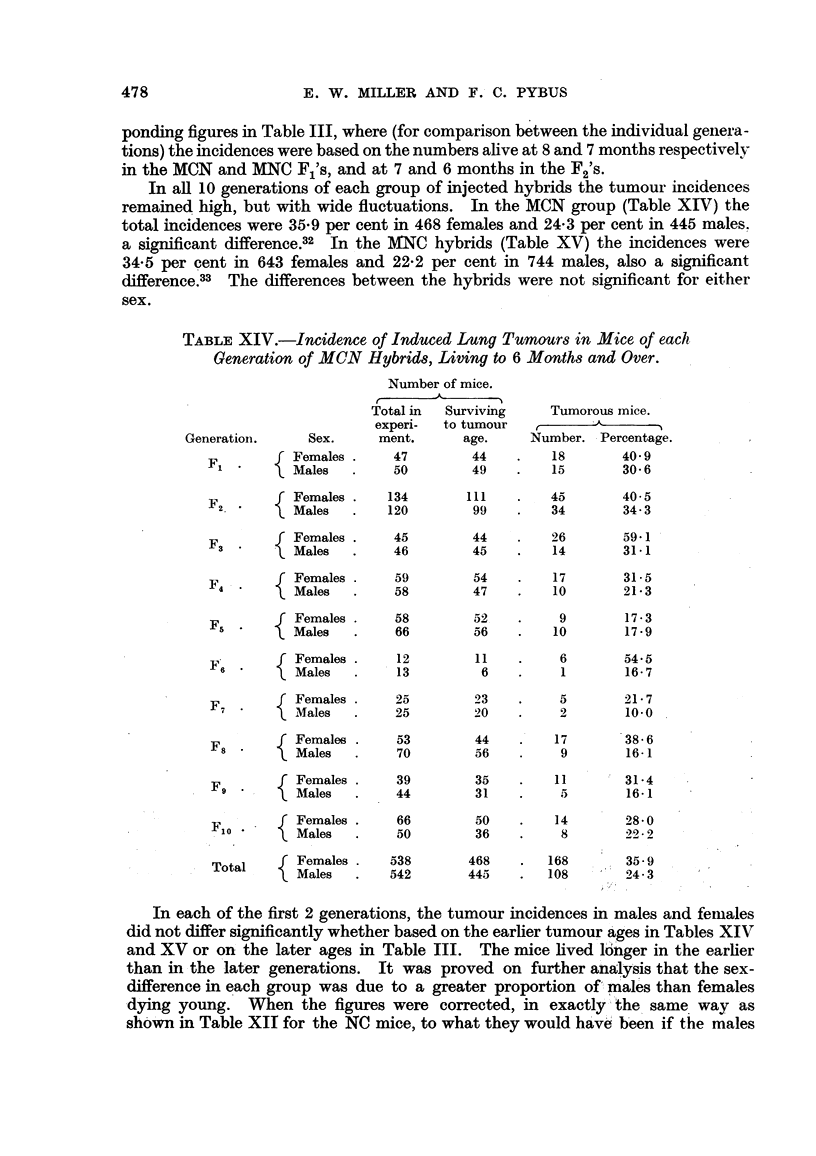

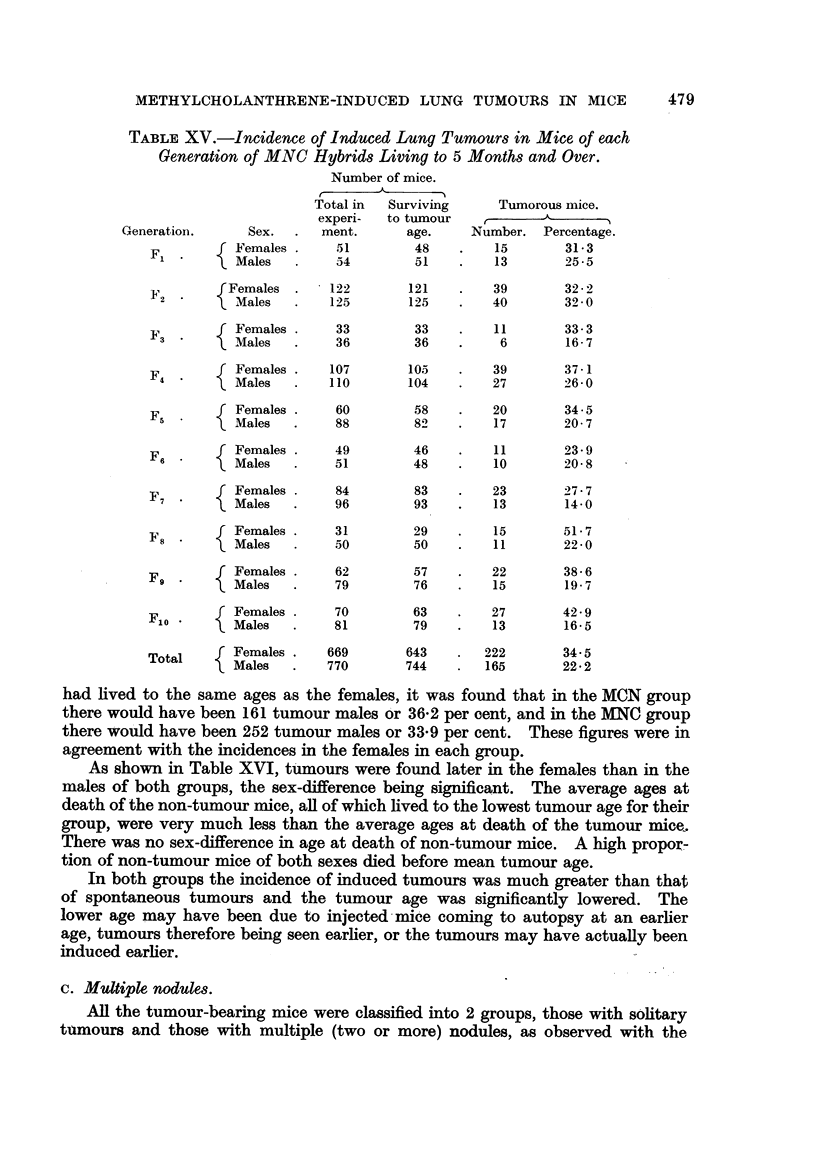

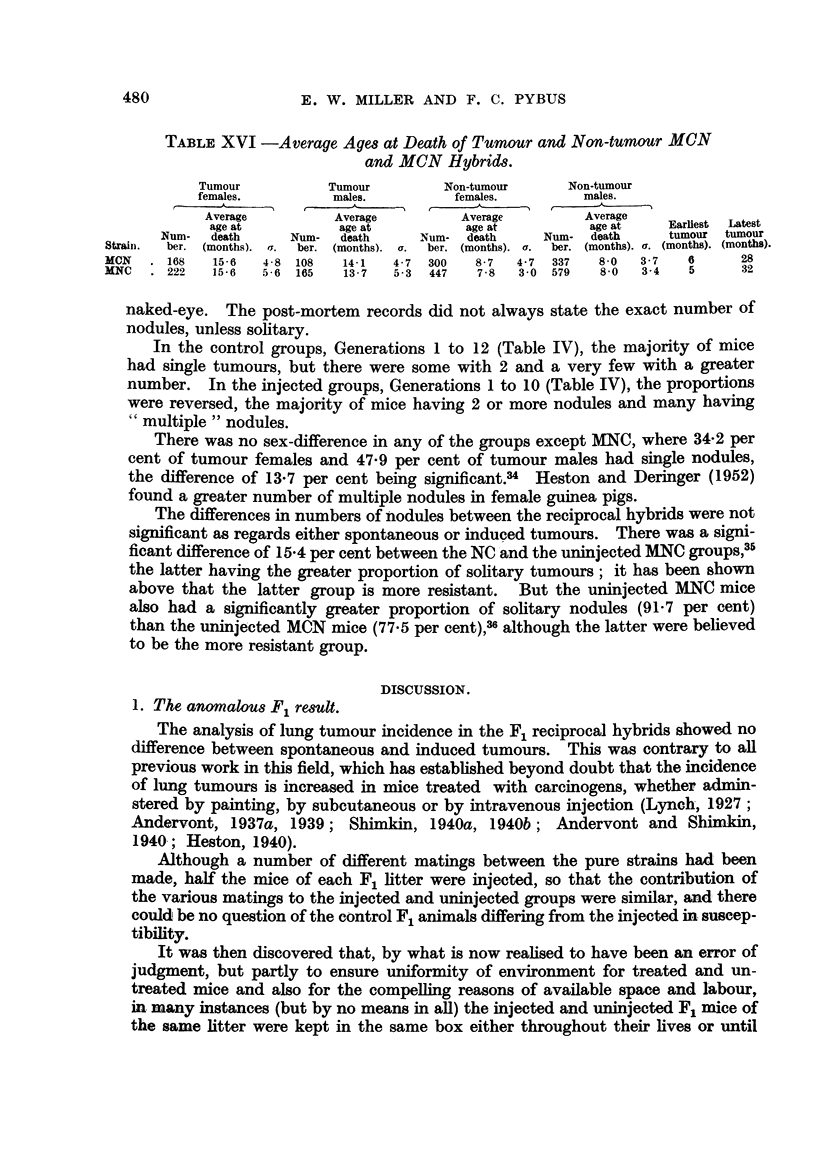

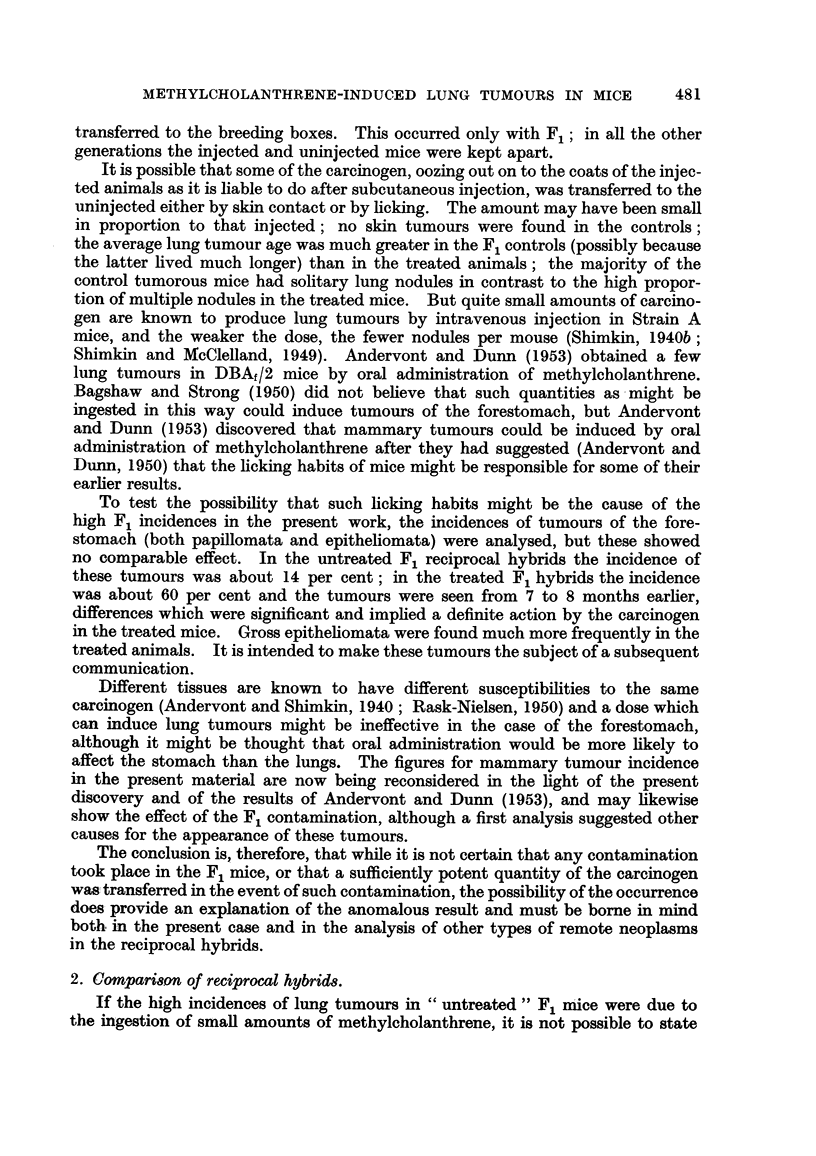

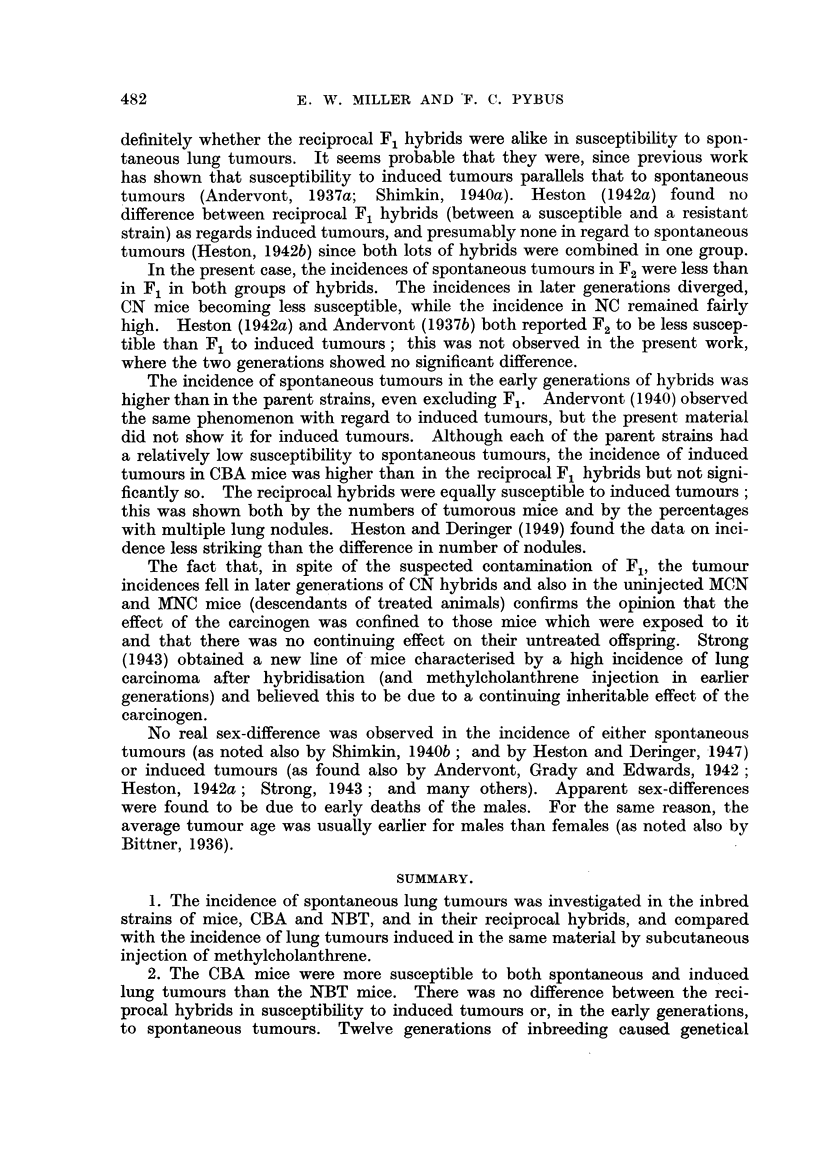

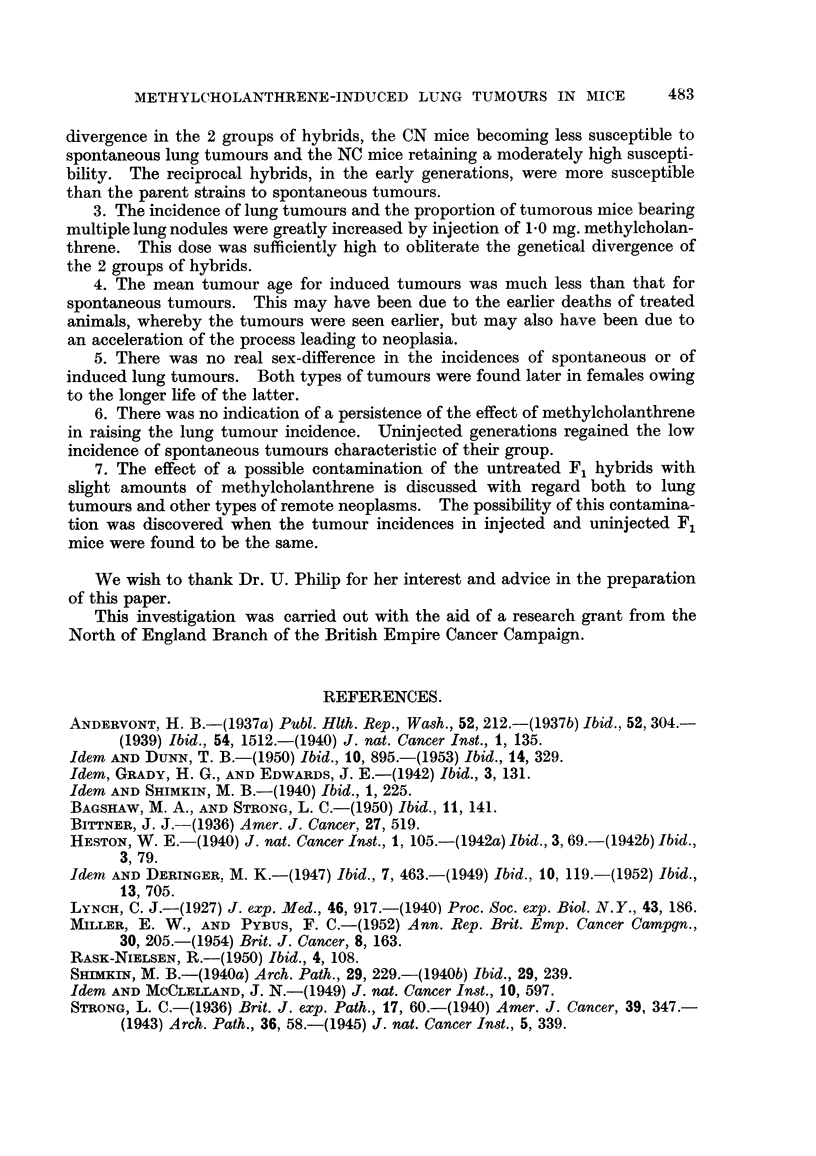

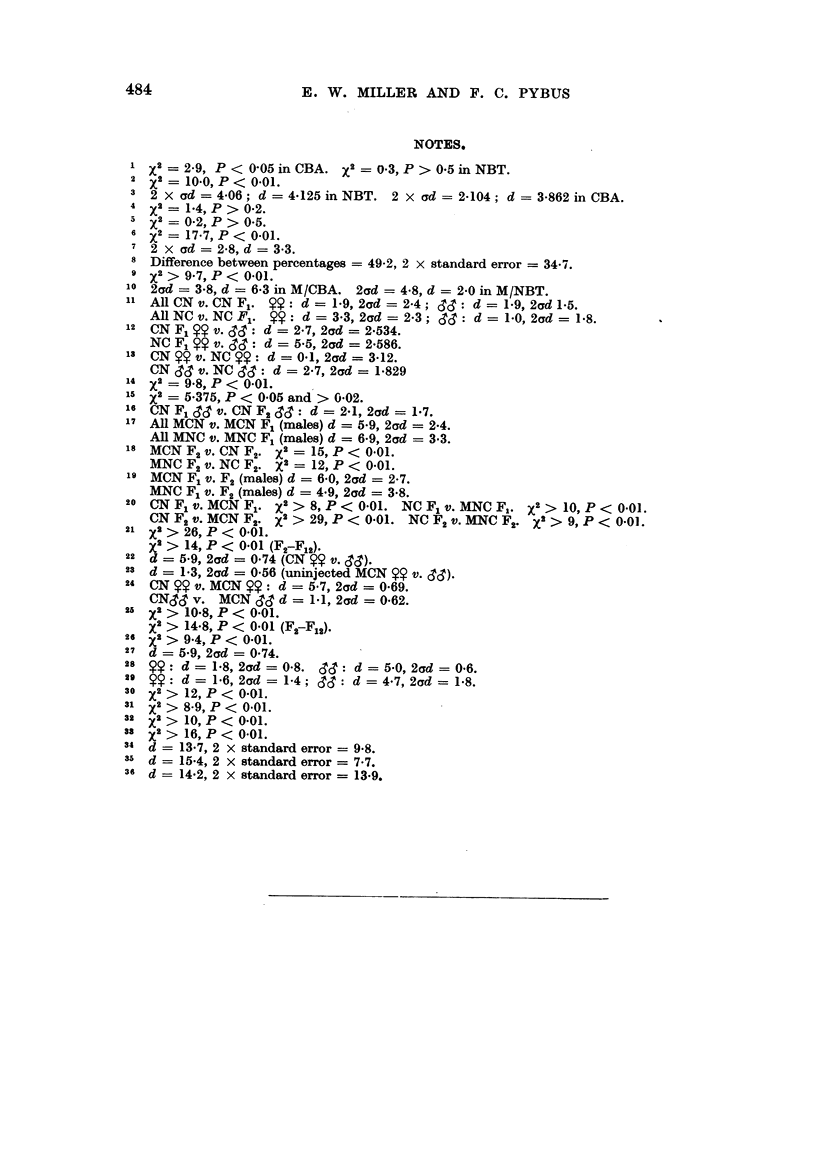


## References

[OCR_01686] BAGSHAW M. A., STRONG L. C. (1950). The occurrence of tumors of the forestomach in mice after parenteral administration of methylcholanthrene: a histopathologic and genetic analysis.. J Natl Cancer Inst.

[OCR_01694] HESTON W. E., DERINGER M. K. (1952). Induction of pulmonary tumors in guinea pigs by intravenous injection of methylcholanthrene and dibenzanthracene.. J Natl Cancer Inst.

[OCR_01697] MILLER E. W., PYBUS F. C. (1954). The local effect of methylcholanthrene on two inbred strains of mice and their reciprocal hybrids.. Br J Cancer.

[OCR_01700] RASK-NIELSEN R. (1950). On the susceptibility of the thymus, lung, subcutaneous and mammary tissues in strain street mice to direct application of small doses of four different carcinogenic hydrocarbons.. Br J Cancer.

[OCR_01705] SHIMKIN M. B., McCLELLAND J. N. (1949). Induced pulmonary tumors in mice; analysis of dose response data with methylcholanthrene.. J Natl Cancer Inst.

